# Dissecting the Role of NF-κb Protein Family and Its Regulators in Rheumatoid Arthritis Using Weighted Gene Co-Expression Network

**DOI:** 10.3389/fgene.2019.01163

**Published:** 2019-11-20

**Authors:** Jamal S. M. Sabir, Abdelfatteh El Omri, Babajan Banaganapalli, Majed A. Al-Shaeri, Naser A. Alkenani, Mumdooh J. Sabir, Nahid H. Hajrah, Houda Zrelli, Lukasz Ciesla, Khalidah K. Nasser, Ramu Elango, Noor Ahmad Shaik, Muhummadh Khan

**Affiliations:** ^1^Center of Excellence in Bionanoscience Research, King Abdulaziz University, Jeddah, Saudi Arabia; ^2^Genomics and Biotechnology Section and Research Group, Department of Biological Sciences, Faculty of Science, King Abdulaziz University, Jeddah, Saudi Arabia; ^3^Department of Genetic Medicine, Faculty of Medicine, King Abdulaziz University, Jeddah, Saudi Arabia; ^4^Biology–Zoology Division, Department of Biological Sciences, Faculty of Science, King Abdulaziz University, Jeddah, Saudi Arabia; ^5^Department of Computer Sciences, Faculty of Computers and Information Technology, King Abdulaziz University, Jeddah, Saudi Arabia; ^6^Department of Biological Sciences, Science and Engineering Complex, The University of Alabama, Tuscaloosa, AL, United States; ^7^Department of Medical Laboratory Technology, Faculty of Applied Medical Sciences, King Abdulaziz University, Jeddah, Saudi Arabia

**Keywords:** rheumatoid arthritis, auto-inflammatory disease, NF-κB, GEO, gene expression

## Abstract

Rheumatoid arthritis (RA) is a chronic synovial autoinflammatory disease that destructs the cartilage and bone, leading to disability. The functional regulation of major immunity-related pathways like nuclear factor kappa B (NF-κB), which is involved in the chronic inflammatory reactions underlying the development of RA, remains to be explored. Therefore, this study has adopted statistical and knowledge-based systemic investigations (like gene correlation, semantic similarity, and topological parameters based on graph theory) to study the gene expression status of NF-κB protein family (NK^PF^) and its regulators in synovial tissues to trace the molecular pathways through which these regulators contribute to RA. A complex protein–protein interaction map (PPIM) of 2,742 genes and 37,032 interactions was constructed from differentially expressed genes (*p* ≤ 0.05). PPIM was further decomposed into a Regulator Allied Protein Interaction Network (RA^PIN^) based on the interaction between genes (5 NK^PF^, 31 seeds, 131 hubs, and 652 bottlenecks). Pathway network analysis has shown the RA-specific disturbances in the functional connectivity between seed genes (*RIPK1*, *ATG7*, *TLR4*, *TNFRSF1A*, *KPNA1*, *CFLAR*, *SNW1*, *FOSB*, *PARVA*, *CX3CL1*, and *TRPC6*) and NK^PF^ members (*RELA*, *RELB*, *NFKB2*, and *REL*). Interestingly, these genes are known for their involvement in inflammation and immune system (signaling by interleukins, cytokine signaling in immune system, NOD-like receptor signaling, MAPK signaling, Toll-like receptor signaling, and TNF signaling) pathways connected to RA. This study, for the first time, reports that SNW1, along with other NK regulatory genes, plays an important role in RA pathogenesis and might act as potential biomarker for RA. Additionally, these genes might play important roles in RA pathogenesis, as well as facilitate the development of effective targeted therapies. Our integrative data analysis and network-based methods could accelerate the identification of novel drug targets for RA from high-throughput genomic data.

## Introduction

Rheumatoid arthritis (RA) is a complex systemic autoinflammatory disorder causing chronic destructive inflammation in synovial joints resulting in severe physical disability in millions of patients worldwide (Dargham et al., 2018). Molecular basis of this disease is not well defined. But, genetic background (polymorphisms or inherited mutations), hormones, cytokines, and environmental factors are believed to be some of the contributing factors (Okada et al., 2019). Among the genetic factors, human leukocyte antigen (HLA) loci (*HLA-DRB1) and non-HLA genes (*PTPN22, PADI4, TRAF1/C5, TNFAIP3, CCR6, REL, etc.) are the main susceptibility risk factors for RA across multiple ethnic groups (Barton et al., 2008; Raychaudhuri et al., 2012; Kochi et al., 2014; Mcgonagle et al., 2018; Shaik and Banaganapalli, 2019). However, these genetic markers, whether single or as a group, are unable to fully explain complex pathogenic mechanisms underlying the disease and are also not robust in acting as systemic biomarkers for diagnosing or monitoring RA.

On the contrary, studying genome-wide gene expression provided a deeper insight into the molecular patterns of different autoimmune diseases (Pimentel-Santos et al., 2011; Rezaei Tavirani et al., 2018; Yin et al., 2019). Gene expression assays provide a quantitative measure of thousands of mRNA molecules in one single experiment. It can also generate a global picture of cell function and also contributes to a stratified medicine application (Smith et al., 2013). But understanding and interpreting the massive amount of genomics data (for around 20,000 genes) generated by high-throughput technologies like microarray-based gene expression is complex and challenging. In recent decades, gene networking based on graph topological parameters and subsequent functional enrichment has revolutionized the disease-centric candidate gene approaches (Sabir et al., 2019). One such highly attractive gene set with strong influence on RA synovial tissues is nuclear factor kappa B (NF-κB) (Noort et al., 2015). Activated NF-κB is detected in human synovial tissues during the early and late stages of inflammation (Gilston et al., 1997).

The NF-κB belongs to a group of transcription factors (NF-κB1, NF-κB2, RelA, RelB, and c-Rel) which controls the expression of many genes involved in immunity and inflammatory processes (Liu et al., 2017). These genes mediate the target gene transcription by binding to NF-κB enhancer, a key DNA element either in hetero- or homo-dimeric form (Oeckinghaus and Ghosh, 2009). Molecular pathways related to the translocation of NF-κB from the nucleus and its function are often strongly regulated, but act in synergy with the activation of NF-κB-dependent gene expression (Makarov, 2001). NF-κB is activated by different pathogenic stimuli, including Toll-like receptors (TLR3, TLR7, TLR8, TL9, TLR1, TLR2, TLR4, and TLR6), growth factors, cytokines, radiation, and oxidative stress. Activated NF-κB is detected in human synovial tissues during both early and late stages of inflammation (Gilston et al., 1997).

In innate immunity, activation of the NF-κB pathway upregulates the expression of defensins, pro-inflammatory proteins like cytokines (IL-1, IL-6, and TNF-α), and proteins involved in leukocyte migration (VCAM1 and ICAM1). In adaptive immunity, NF-κB is involved in the proliferation of B- and T-cells as well as in the maturation of dendritic cells (Verma et al., 2019). Although NF-κB is ubiquitous and rigorously studied, some questions about this pathway including transcription machinery, stimulus-specific gene expression, and cell types are yet to be explored. Keeping in view of the above-described facts, this study has adopted statistical and knowledge-based systemic investigations (like gene correlation, semantic similarity, and topological parameters based on graph theory) to study the expression status of NF-κB regulators in synovial tissues and to trace the molecular pathways through which these regulators contribute to RA.

## Materials and Methods

### Selection of NF-κb Regulators

The top 20 known NF-κB pathway regulators controlling the immune response to independent TNF-α and lipopolysaccharide (LPS) antigenic treatments were identified from a large-scale secondary RNAi screening on differentiated human THP-1 monocyte cell lines (Verma et al., 2019). These top 40 NF-κB regulators were chosen based on the significant reductions in luciferase activity of their siRNAs upon both TNF-α and LPS treatments and did not cause significant loss of cell viability *in vitro*. For ease of description, regulators of NF-κB protein family are termed as “seed genes” and the NF-κB protein family as “NK^PF^.”

### Microarray Gene Expression Data

The microarray gene expression profile of RA patients’ synovial tissues with series identifier GSE77298 was taken from the GEO database (www.ncbi.nlm.nih.gov/geo). Total number of samples in the dataset was 23 (**Table S1**). Gene expression data was generated with U133Plus 2.0 oligonucleotide array (Affymetrix, Santa Clara, CA, USA) from 16 end-stage RA synovial biopsies and seven synovial biopsies from healthy individuals without a joint disease (healthy control, HC) (Broeren et al., 2016). Genes with a fold change threshold of 1.2 (−1.2 ≥ FC ≥ +1.2) with *P* value ≤ 0.05 in RA vs. HC were selected from the analysis. Information about the patient samples are given in the supplementary file (**Supplementary Table 1**).

### Data Normalization and Analysis

Analysis of microarray gene expression data was carried out by using R/Bioconductor (Carvalho and Irizarry, 2010; Ritchie et al., 2015). For the standardization and noise reduction of the probe data, CEL files were loaded into R package-*Affy* and raw signal values for each probe sets were normalized. Normalization of the microarray dataset was performed using Robust Multiarray Average (RMA) algorithm (Carvalho and Irizarry, 2010). Statistically significant differentially expressed genes between normal and RA samples were computed by applying *t*-statistic. False discovery rate (FDR) of Benjamini and Hochberg with *p* value ≤ 0.05 was applied on the significant gene data to remove false positives (Benjamini and Hochberg, 2001).

### Protein-Protein Interaction Mapping

An experimentally validated protein–protein interaction map (PPIM) was constructed using a Cytoscape plugin, Bisogenet, which extracts the interaction among queried genes from the data deposited in the Biomolecular Interaction Network Database (BIND), Biological General Repository for Interaction Datasets (BioGRID), The Molecular Interaction Database (MINT), Database of Interacting Proteins (DIP), Human Protein Reference Database (HPRD), and IntAct database (Xenarios et al., 2000; Bader et al., 2003; Chatr-Aryamontri et al., 2007; Keshava Prasad et al., 2009; Aranda et al., 2010; Chatr-Aryamontri et al., 2017). Selected differentially expressed genes (DEGs) from the microarray data are used as input in Bisogenet to generate PPIM (Sabir et al., 2019). Construction of the interactome was built from the DEGs. The output is in the form of graph, which represents gene as node and interaction between genes as edge (Martin et al., 2010; George et al., 2019).

### Construction of Sub-Network

A sub-network of Regulator Allied Protein Interaction Network (RA^PIN^) was constructed from PPIM by implementing well-established theories like degree centrality (DC) and betweenness centrality (BC) in network biology. From the PPIM, we identified those genes that fit to: a) hubs which are dependent on DC, b) bottlenecks based on BC, and c) NF-κB proteins and regulators. The centrality parameters or network properties were scaled using ‘*Network Analyzer*’, a Cytoscape plugin (Shannon et al., 2003; Assenov et al., 2008).

a) Identification of Hub Proteins

We used a method developed by Rakshit et al. (2014) to detect the hubs in the biological network. The formula for picking hubs is as follows:

(Eq. 1) Hubs=Avg(DC)+[2×SD(DC)]

where Avg is the mean DC of all genes in the biological network PPIM and SD denotes their standard deviation.

b) Identification of Bottlenecks

BC was introduced to scale the bottleneck genes in the interactome. The formula for calculating BC is as follows:

(Eq. 2) $$\mathrm{BC}(n)=\sum_{s\neq n\neq t}[\frac{\sigma_{\mathrm{st}}(n)}{\sigma_{\mathrm{st}}}]$$

where *s* and *t* are nodes in the network other than *n*, σ*_st_* represents the number of shortest paths from *s* to *t*, and σ*_st_*(*n*) is the number of shortest paths from *s* to *t* that *n* lies on. Genes located in the top 25% of betweenness were extracted as bottleneck genes.

### Building of Weighted Correlation Map

Pearson’s correlation algorithm was applied to the genes of RA^PIN^ to create a weighted gene correlation map. The Pearson’s correlation coefficient (PCC) of pairs of genes is measured using the following formula:

(Eq. 3) $$\mathrm{PCC}(r)=\frac{\sum_{i=1}^{n}(x_{i}-\underline{x})\left(y_{i}-\underline{y}\right)}{\sqrt{\sum_{i=1}^{n}{\left(x_{i}-\underline{x}\right)}^{2}}\sqrt{\sum_{i=1}^{n}{\left(y_{i}-\underline{y}\right)}^{2}}}$$

where *x* and *y* are the averages of sample expression values in healthy and RA conditions of the two genes, respectively.

### Functional Similarity Between Gene Pairs

Functional resemblance among two genes is evaluated using prearranged data available in Gene Ontology. To evaluate the functional similarity between two genes, Wang’s measure of semantic similarity was applied to molecular function (MF) hierarchy as MF, which specifically defines a particular gene in terms of functional ontology. The semantic score of functional similarity between genes range from 0 to 1. Higher semantic score between genes represents a stronger functional relationship among the genes (Wang et al., 2007). The semantic score of functional similarity between gene pairs is measured as follows;

(Eq. 4) $$S_{\mathrm{GO}}(X,Y)=\frac{\sum_{t\in T_{X}\cap^{\text{​}}T_{Y}}(S_{X}(t)+S_{Y}(t))}{\sum_{t\in T_{X}}S_{X}(t)+\sum_{t\in T_{Y}}S_{Y}(t)}$$

where *T* *_X_* is the set of all its ancestor ontology as well as ontology *X* itself and *S* *_X_*(*t*) represents the contribution of a term *t* ∈ *T* *_X_* to the semantics of *X* based on the relative locations of *t* and *X* in the graph. A single gene can be annotated by multiple gene ontology (GO) relationships. Best-match average (BMA) approach was implemented integrating semantic similarity of multiple GO annotations and evaluates the mean of all maximum similarities. Based on this model, we used R package, *GoSemSim* (Yu et al., 2010), to quantify the semantic similarity between co-expressed gene pairs.

### Functional Enrichment Analysis

Functional annotation is performed to gain insights into the high-throughput biological data. This method not only authenticates the new genes found in biological experiment as functionally significant but also uncovers the biological interactions among them. We used ToppGene Suite to conduct functional enrichment analysis of the filtered gene sets (Chen et al., 2009). The input for ToppGene Suite is the list of DEGs that are identified from gene expression profiles. We applied parameters of gene limits ranging from 2 (minimum interaction) to maximum with statistical significance of *p* ≤ 0.01 and FDR less than 0.05. This suite retrieves information from many public databases which identify key biological processes, cellular components, molecular functions, diseases, and biological pathways.

### GWAS Comparative Analysis

Genome-wide association studies (GWAS) are a dominant and broadly used tool to map the susceptibility loci, genes, and genetic markers in complex traits and diseases. GWAS based on pathway analysis is extensively used to unravel novel multigenic functional relationships. In this study, an attempt was made to explore the pathways commonly enriched between RA-GWAS catalog (https://www.ebi.ac.uk/gwas/) and co-expressed seed genes in our dataset (Macarthur et al., 2017). The list of filtered co-expressed genes was mapped against the data in the GWAS database with p ≤ 0.01. Further, we extracted the reported traits of these co-expressed genes from GWAS catalog to identify their association to RA.

## Results

### Selected NF-κb Regulators

**Figure 1:**
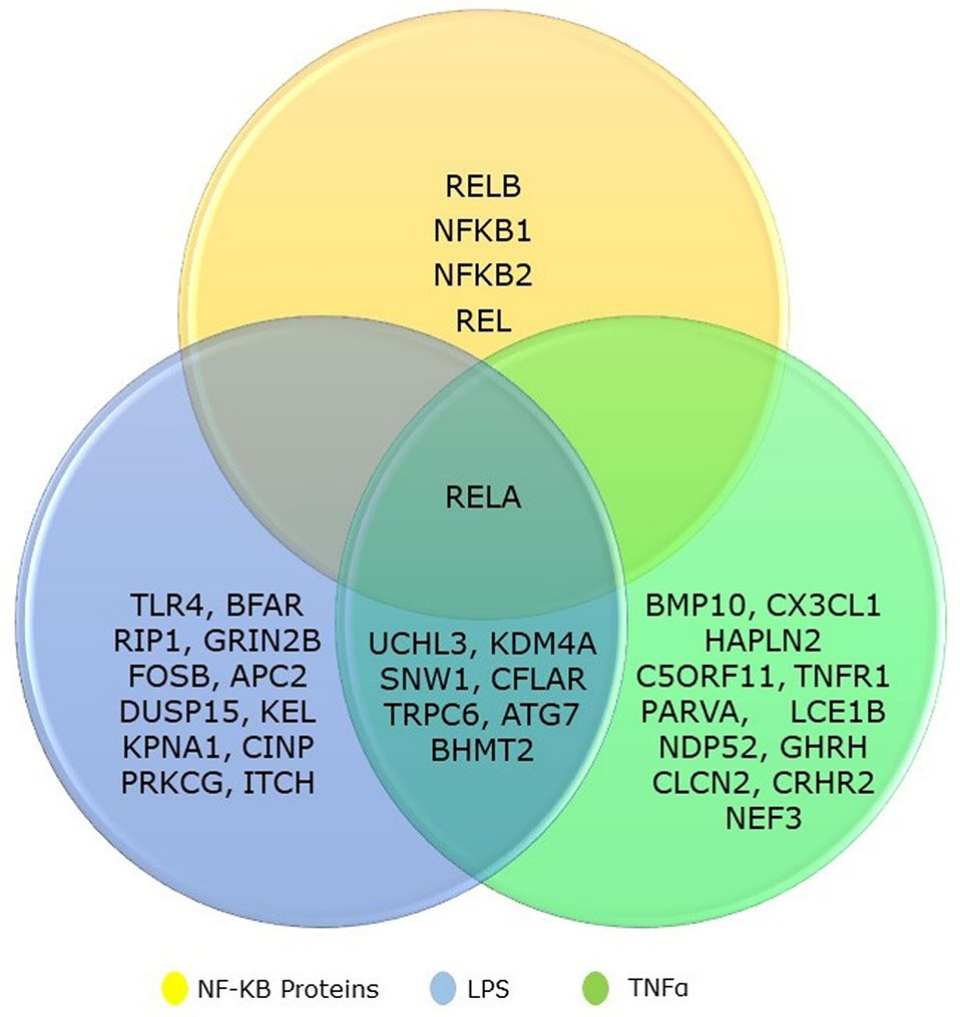
Venn diagram showing the overlapping between NF-kB gene family and its regulatory genes identified from TNFα and LPS secondary screen analysis.

We collected the top 20 hits each from TNF-α and LPS secondary screen analysis and the five genes from NF-κB family. We identified that eight genes are common to both TNF-α and LPS secondary screen analysis and gene *RELA* was part of all three categories (**Figure 1**). Hence, the collected genes for our analysis included 31 seed genes and five genes from NK^PF^ without any redundancy. The list of the selected 36 genes for the analysis is in **Table 1**.

**Table 1:** List of seed and NK^PF^ genes screened in RA samples.

S. no.	Gene symbol	Gene name
NK^PF^		
1.	NFKB1	Nuclear factor kappa B subunit 1
2.	NFKB2	Nuclear factor kappa B subunit 2
3.	REL	Rel proto-oncogene, NF-κB subunit
4.	RELA	Rela proto-oncogene, NF-κB subunit
5.	RELB	Relb proto-oncogene, NF-κB subunit
Seed genes		
6.	CLCN2	Chloride voltage-gated channel 2
7.	CRHR2	Corticotropin-releasing hormone receptor 2
8.	FOSB	Fosb proto-oncogene, Ap-1 transcription factor subunit
9.	GHRH	Growth hormone-releasing hormone
10.	GRIN2B	Glutamate ionotropic receptor NMDA type subunit 2B
11.	KEL	Kell metallo-endopeptidase
12.	KPNA1	Karyopherin subunit alpha 1
13.	NEFM	Neurofilament medium
14.	PRKCG	Protein kinase C gamma
15.	CX3CL1	C-X3-C motif chemokine ligand 1
16.	TLR4	Toll-like receptor 4
17.	TNFRSF1A	TNF receptor superfamily member 1A
18.	TRPC6	Transient receptor potential cation channel subfamily C member 6
19.	UCHL3	Ubiquitin C-terminal hydrolase L3
20.	RIPK1	Receptor interacting serine/threonine kinase 1
21.	CFLAR	Casp8 and FADD-like apoptosis regulator
22.	KDM4A	Lysine demethylase 4A
23.	CALCOCO2	Calcium binding and coiled-coil domain 2
24.	APC2	APC regulator of Wnt signaling pathway 2
25.	ATG7	Autophagy related 7
26.	SNW1	SNW domain containing 1
27.	BHMT2	Betaine–homocysteine S-methyltransferase 2
28.	BMP10	Bone morphogenetic protein 10
29.	BFAR	Bifunctional apoptosis regulator
30.	CINP	Cyclin-dependent kinase 2 interacting protein
31.	PARVA	Parvin alpha
32.	HAPLN2	Hyaluronan and proteoglycan link protein 2
33.	ITCH	Itchy E3 ubiquitin protein ligase
34.	DUSP15	Dual specificity phosphatase 15
35.	LIX1	Limb and CNS expressed 1
36.	LCE1B	Late cornified envelope 1B

### Pre-Processed Data and DEGs

**Figure 2:**
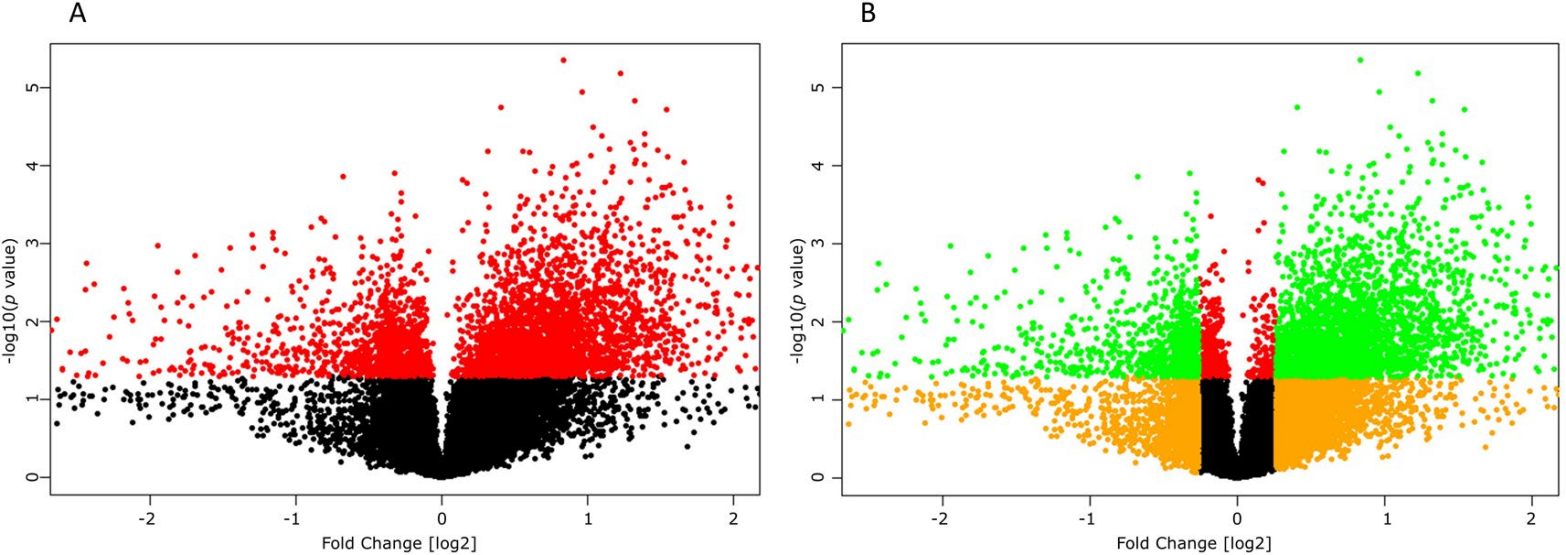
Volcano plots depicting significant differentially expressing genes (DEGs). **(A)** Plot displaying the significant (*red*, *p* ≤ 0.05) and non-significant (*black*, *p* ≥ 0.05) genes. **(B)** Plot displaying the DEGs (*green*, −1.2 ≥ FC ≥ +1.2) and non-significant (*orange*, −1.2 ≤ FC ≤ 1.2) genes.

**Figure 3:**
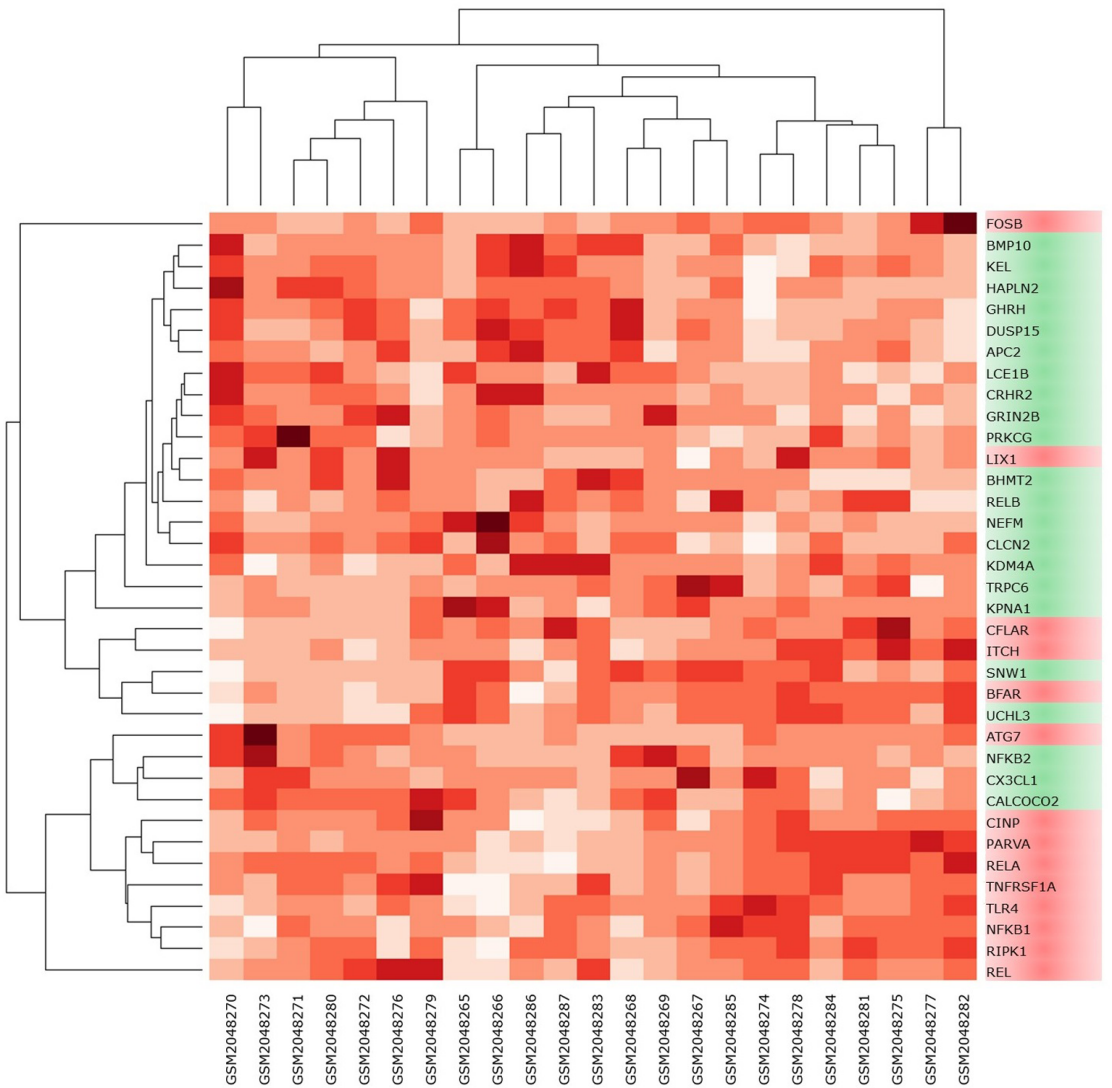
Heat map of NK^PF^ and seed genes in individual sample. The darker the square, the higher the gene expression in that sample.

High-throughput experimental gene expression profiles of synovial tissues, which are affected in RA, were retrieved from the GEO database. The raw signal intensities of 45,056 probes were standardized by means of RMA algorithm, which bring about 3,573 non-redundant DEGs with a statistically significant *p* ≤ 0.05 and fold change of 1.2 (−1.2 ≥ FC ≥ +1.2). The normalization plot of standardized genes is given in **Supplementary File 1** (**Figure S1**). The DEGs comprise 2,577 upregulated and 996 downregulated genes. The volcano plot representing the separation of significant DEGs is depicted in **Figure 2**. Gene expression profiles of seed genes and NK^PF^ extracted and depicted in **Figure 3**.

**Figure 4:**
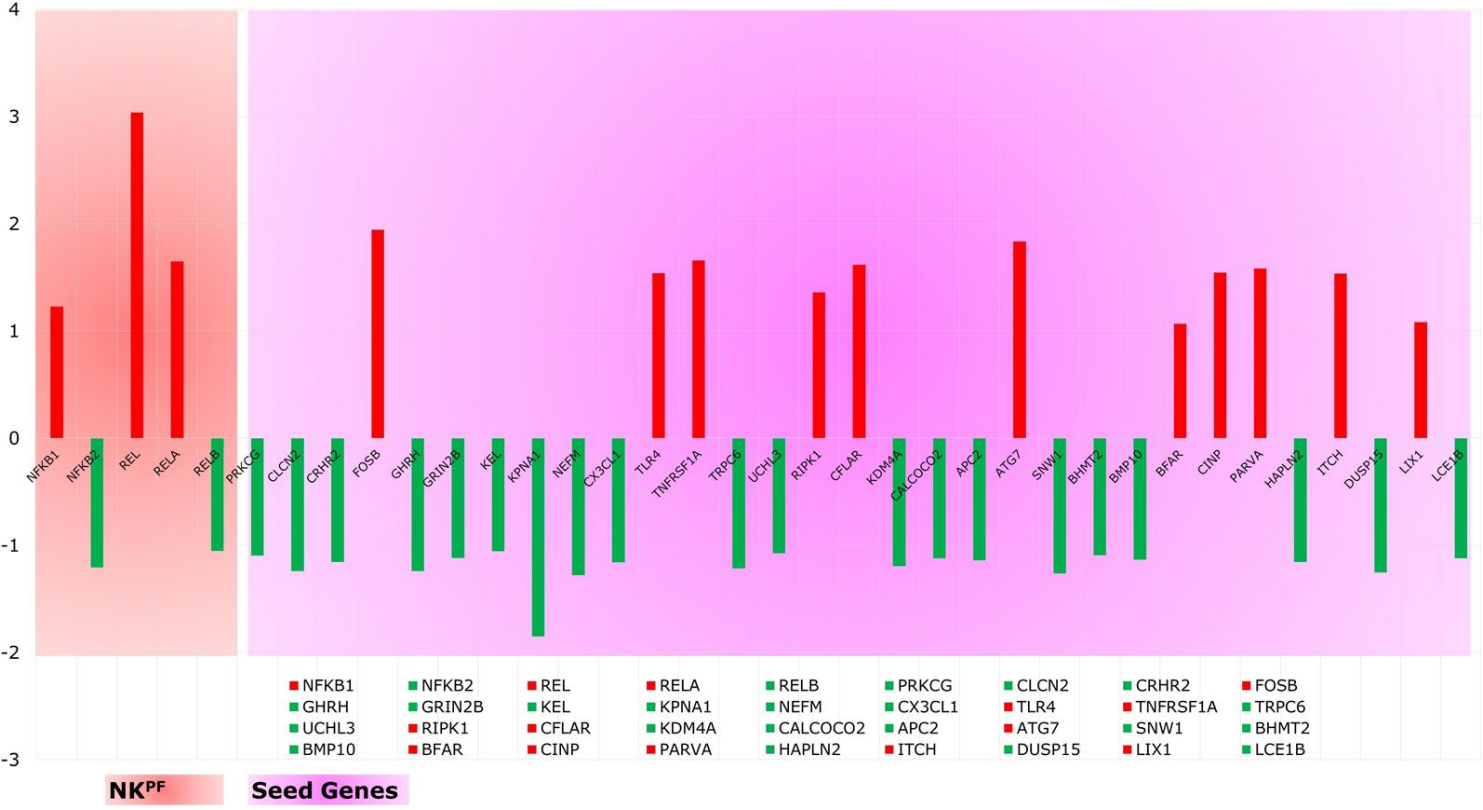
Expression profile of NK^PF^ and seed genes. *Red* and *green* colors are the up- and downregulated genes, respectively.

Analysis of extracted seed genes and NK^PF^ profiles revealed that majority of the seed genes are downregulated. Overall, of 36 genes, 14 are upregulated and 22 are downregulated. **Figure 4** shows the expression of each gene in the sample. Among the NK^PF^ category, *NFKB1*, *REL*, and *RELA* are found to be upregulated, with FC ≥ 1.2 in the RA sample, whereas *NFKB2* and *RELB* are downregulated, with FC ≤ −1.2. The most upregulated gene is *REL*, with FC of 3.04, and the most downregulated gene is *KPNA1*, with FC of −1.85. It is interesting to note that the most upregulated gene, *REL*, falls into the NK^PF^ group, whereas most downregulated gene, *KPNA1*, comes from the seed gene category.

### The Protein–Protein Interaction Map

Significant DEGs, 3,573 genes, from the microarray data were used in Bisogenet to build PPIM by extracting all relationships between the queried genes. The PPIM was made stable by eliminating self-loops and repeated edges to calculate the standardized centrality parameters of the whole interactome. Bisogenet produced a complex PPIM which included 2,742 genes and 37,032 interactions, with 13.51 mean edge–node fraction, as given in the supplementary file (**Supplementary File 2**, **Figure S1**). The Cytoscape “Network Analyzer” plugin was employed to measure the DC and BC of the interactome.

### Regulator Allied Protein Interaction Network

The DEGs in the PPIM were classified into bottlenecks and hubs based on centrality properties for the construction of RA^PIN^. The threshold cutoff value for bottlenecks (based on BC) and hubs (based on DC) were set based on Eqs. (2) and (1), respectively. A total of 652 genes were categorized as bottlenecks and 131 genes as hubs. Interestingly, seed and NK^PF^ genes like *NFKB1*, *REL*, *RELA*, *TLR4*, *TNFRSF1A*, and *SNW1* were in the category of hubs and *GRIN2B*, *KPNA1*, *NFKB2*, *PRKCG*, *RELB*, *CX3CL1*, *RIPK1*, *CFLAR*, *CALCOCO2*, *ATG7*, and *ITCH* were detected in the bottleneck group (**Table 2**). The hubs, bottlenecks, NK^PF^, and seed genes together constitute non-redundant 801 nodes. Interactions among those 801 genes were extracted from the main PPIM to construct regulator specific subnetwork, RA^PIN^ (**Supplementary File 2**, **Figure S2**). Subnetwork RA^PIN^ encompasses 801 nodes and 19,050 edges, with 23.75 average edge–node ratio.

**Table 2:** The NK^PF^ and seed genes in the hub and bottleneck categories.

	Symbol	Gene name	BC	DC
Hubs				
1.	SNW1	SNW domain containing 1	1.18 × 10^−02^	170
2.	TLR4	Toll-like receptor 4	6.29 × 10^−03^	170
3.	RELA*	RELA proto-oncogene, NF-κB subunit	7.91 × 10^−03^	151
4.	NFKB1*	Nuclear factor kappa B subunit 1	4.86 × 10^−03^	142
5.	TNFRSF1A	TNF receptor superfamily member 1A	3.26 × 10^−03^	123
6.	REL*	REL proto-oncogene, NF-κB subunit	8.09 × 10^−03^	100
Bottlenecks				
1.	ATG7	Autophagy related 7	4.38 × 10^−03^	85
2.	ITCH	Itchy E3 ubiquitin protein ligase	3.81 × 10^−03^	83
3.	RIPK1	Receptor interacting serine/threonine kinase 1	1.18 × 10^−03^	68
4.	NFKB2*	Nuclear factor kappa B subunit 2	9.26 × 10^−04^	67
5.	RELB*	RELB proto-oncogene, NF-κB subunit	1.02 × 10^−03^	65
6.	GRIN2B	Glutamate ionotropic receptor NMDA type subunit 2B	1.23 × 10^−03^	56
7.	CFLAR	CASP8 and FADD-like apoptosis regulator	1.03 × 10^−03^	51
8.	PRKCG	Protein kinase C gamma	6.70 × 10^−04^	48
9.	CX3CL1	C-X3-C motif chemokine ligand 1	2.23 × 10^−04^	48
10.	CALCOCO2	Calcium binding and coiled-coil domain 2	1.52 × 10^−03^	44
11.	KPNA1	Karyopherin subunit alpha 1	8.64 × 10^−04^	39

*Genes from NK^PF^.

### Co-Expression Pattern Among the RA^PIN^ Genes

**Figure 5:**
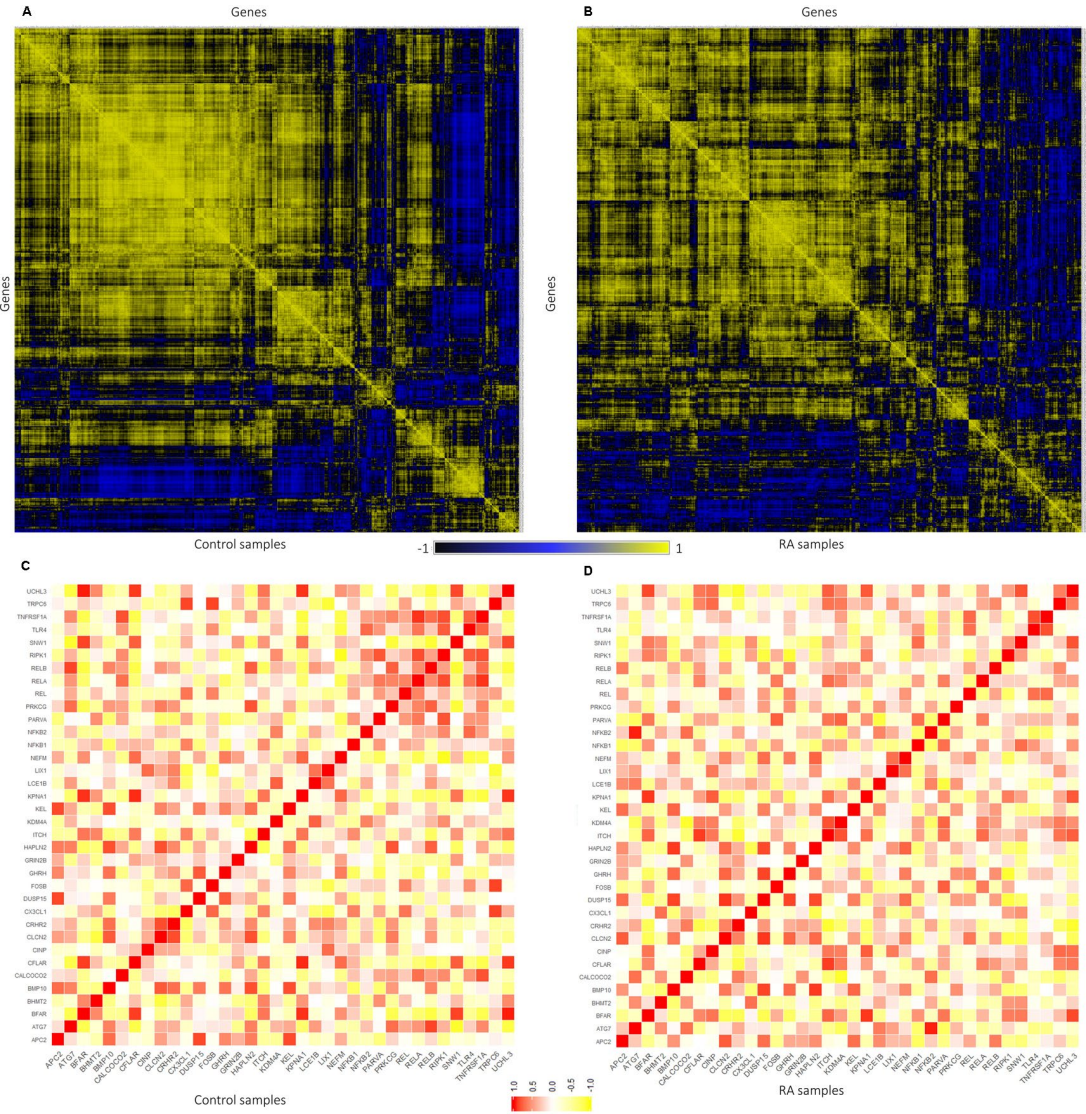
Representation of gene–gene correlation plots for healthy and rheumatoid arthritis (RA) samples. **(A)** Correlation of gene pairs in control samples (normal). **(B)** Correlation of gene pairs in RA samples. **(C)** Gene–gene correlation of NK^PF^ and seed genes in control samples. **(D)** Gene–gene correlation of NK^PF^ and seed genes in RA samples.

The expression level similarity across 801 genes of RA^PIN^ was created and ranked for both disease and sample data sets using Pearson’s correlation algorithm (**Figure 5**). Pearson’s correlation algorithm applied to the gene sets created PCC for 320,400 gene pairs from 801 genes belonging to both disease and control samples (Eq. 3). Here, we selected pairs of genes using the following well-established concepts. 1) Expression of gene pairs with high positive correlation. 2) Genes of similar patterns of expression are more likely to interact with one another. Gene pairs with *r* ≥ 0.8 from the correlation map were chosen for the analysis for both healthy and disease, as a higher *r* score represents stronger association (Rakshit et al., 2014). Corresponding gene pairs were extracted from the normal and RA correlation map to identify the variation in the co-expression from control to affected tissues.

**Figure 6:**
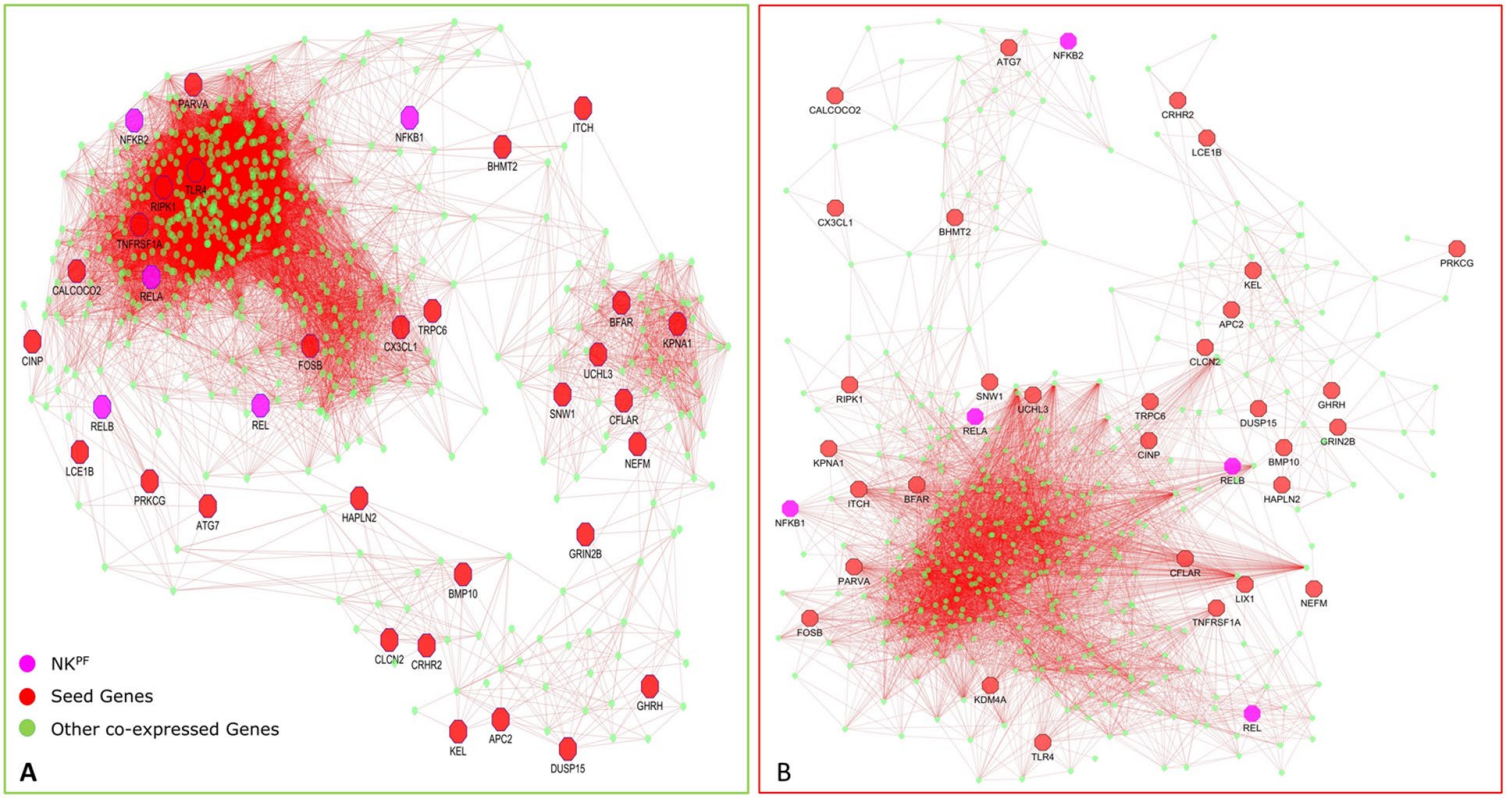
Network of co-expressed RA^PIN^ genes in healthy controls **(A)** and in RA **(B)**.

In normal samples, 601 genes were found to have higher correlation and 461 were co-expressed in disease samples (**Figure 6**). Interestingly, all seed genes were co-expressed in the RA sample, whereas seed genes *KDM4A* and *LIX1* were not co-expressed in normal samples. The total number of gene pairs with higher correlation in normal condition was 28,350 and in RA was 8,747. The correlation loss from normal to RA condition indicates a functional disruption at the genome level.

**Figure 7:**
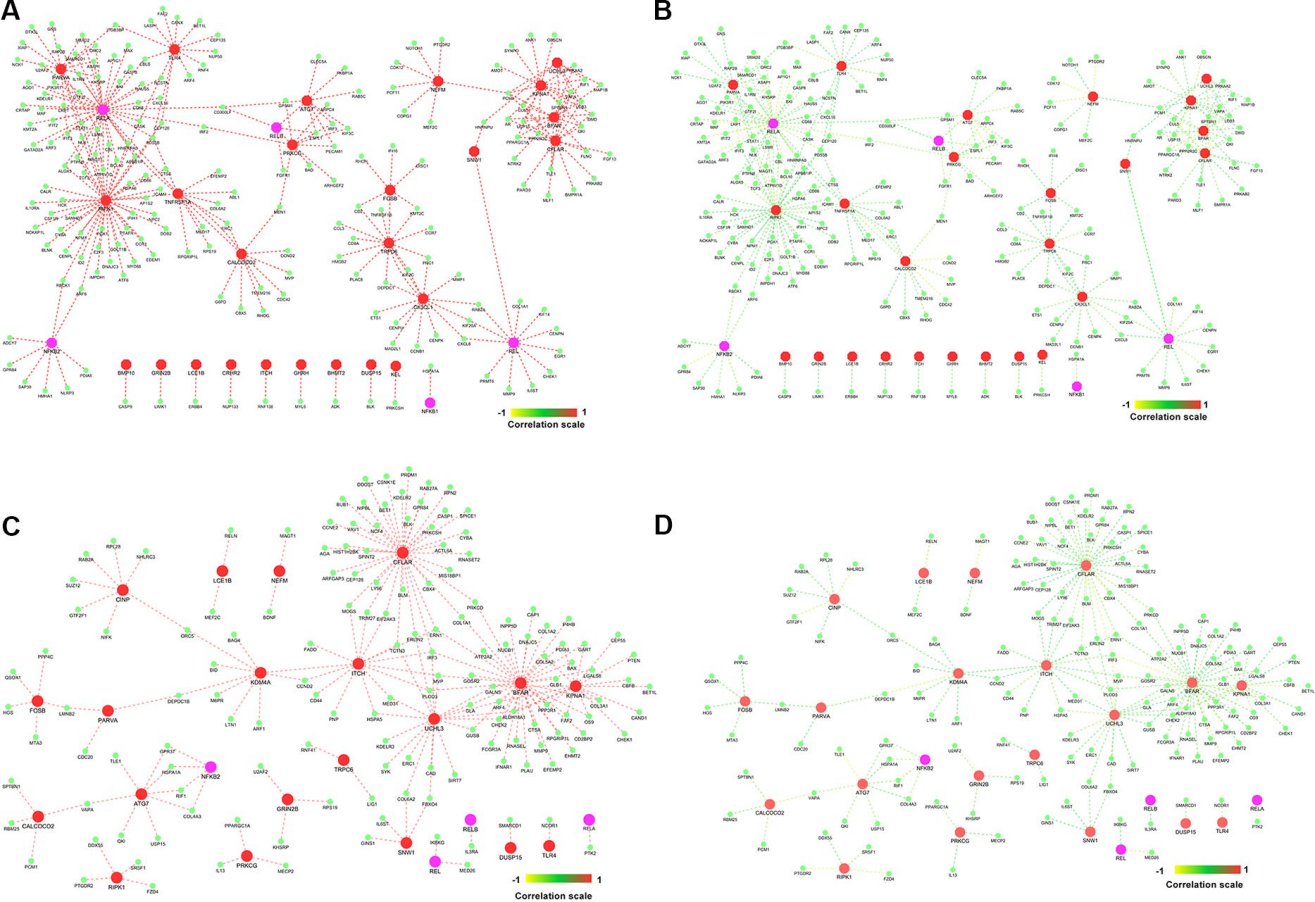
Gene–gene correlation of seed genes, NK^PF^, and their partners. **(A)** Co-expression of gene pairs in normal condition. **(B)** Corresponding correlation of same gene pairs (in **A**) in rheumatoid arthritis (RA). **(C)** Co-expression of gene pairs in RA. **(D)** Corresponding correlation of gene pairs (in **C**) in healthy controls.

Highly co-expressed 28,350 gene pairs in normal samples were mapped to RA to extract the correlation of the same gene pairs to understand the critical variation from normal to RA. Similarly, 8,747 co-expressed gene pairs of RA samples were mapped to normal samples. The absolute difference of correlation was calculated for the gene pairs in both normal and RA conditions. The value of absolute difference ranged from 0 to 2. As the difference value increases, distinction of these gene pairs also increases from one condition to another. Next, we focused on the genes interacting with seed genes and NK^PF^ with distinct variation. We considered a cutoff absolute value of 0.5 to select the gene pairs with distinct variations. We obtained 746 gene pairs (476 genes) in the normal condition that have critical variation of expression from RA. Similarly, 453 gene pairs (318 genes) in RA had distinct deviation from normal condition. Further, we extracted only the connectivity of seed genes and NK^PF^ from the aforesaid interactome. We found 227 genes co-expressed with NK^PF^ and seed genes in normal condition and 171 co-expressed genes in RA condition (**Figure 7**). We observed a drastic change of connectivity among NF-κB family of proteins with NF-κB regulators (genes from TNF-α and LPS antigenic treatment) from normal to RA condition, which clearly indicates disruption of the NF-κB signaling pathway.

### Semantic Similarity Between Co-Expressed Gene Pairs

**Figure 8:**
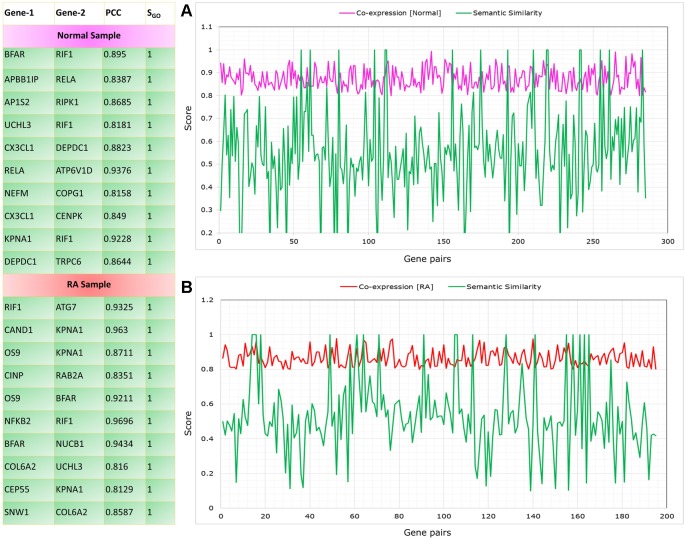
Top gene pairs with their corresponding correlation and semantic scores for both control and rheumatoid arthritis (RA) samples. The graph shows correlation and semantic scores for significantly correlated gene pairs in control **(A)** and RA samples **(B)**.

The co-expressed gene pairs were selected from the RA^PIN^ and semantic similarity of Wang’s measure was applied (Wang et al., 2007). Functional similarity was generated based on the semantic score for co-expressed gene pairs by implementing *GoSemSim* module in R. **Figure 8** depicts the list of top 10 gene pairs with their corresponding correlation and semantic scores for both control and RA samples. Next, we filtered 203 gene pairs (156 genes) from normal condition and 115 gene pairs (101 genes) from RA condition with robust functional relationship depending on a semantic score of ≥0.5.

In normal condition, the seed genes are directly or indirectly connected to NF-κB protein family with high correlation, whereas there is a serious disruption in the connectivity of NK^PF^ with seed genes or *vice versa* in RA. To pinpoint the dysregulation among NK^PF^ and seed genes, we performed functional enrichment of connected genes in the normal sample.

### Functional Enrichment of Co-Expressed Genes

Gene set enrichment analysis of interconnected genes in the normal sample was performed using ToppGene Suite. The gene sets were enriched in few molecular functions, biological processes, cellular components, and pathways. Fifty-four genes fall into the category of “enzyme binding” (FDR = 8.57 × 10^−18^), 47 in the category of “protein-containing complex binding” (FDR = 7.59 × 10^−16^), and nine in the category of “identical protein binding” (FDR = 1.82 × 10^−07^), which were the top 3 terms of GO molecular function (MF). The top 10 MF among the enriched genes are listed in **Table 3**. GO cellular component (CC) enrichment showed that the gene products mainly are in “cytoskeletal part” (FDR = 9.60 × 10^−09^), with a count of 34 genes, followed by “cell junction” (FDR = 7.13 × 10^−09^) and “neuron part” (FDR = 5.23 × 10^−06^), with gene counts of 30 and 29, respectively. The top 10 CC among the enriched genes are represented in **Table 4**. As to the biological process (BP) enrichment, “programmed cell death” (FDR = 1.39 × 10^−15^), “positive regulation of gene expression” (FDR = 1.76 × 10^−13^), and “immune response” (FDR = 4.48 × 10^−15^) were the most annotated terms, with gene counts of 51, 47, and 45, respectively. The top 10 BP enriched terms are listed in **Table 5**.

**Table 3:** Top 10 molecular functions enriched with their respective gene ontology terms and genes.

No.	GO ID	Name	*p* value	Count	Genes
1	GO:0019899	Enzyme binding	8.57 × 10^−18^	54	*NPM1*, *NUP50*, *TCF3*, *ABL1*, *ANK1*, *RIPK1*, *CALR*, *ERC1*, *SPTBN1*, *TRPC6*, *BAD*, *BLNK*, *ETS1*, *U2AF2*, *XIAP*, *RPS19*, *NPC2*, *RELA*, *NCK1*, *BFAR*, *PRC1*, *SKI*, *CBL*, *CBLB*, *AR*, *STAT1*, *RBCK1*, *CCNB1*, *ARF6*, *CCND2*, *KIF20A*, *CUL5*, *CFLAR*, *LDB3*, *GTF2I*, *AGO1*, *CSF1R*, *FAF2*, *RNF4*, *AP1G1*, *EGR1*, *CBX5*, *PIK3R1*, *LRP1*, *BCL10*, *DMD*, *TNFRSF1A*, *CDC42*, *FKBP1A*, *MVP*, *HSPA6*, *NOTCH1*, *NLK*, *SMAD2*
2	GO:0044877	Protein-containing complex binding	7.59 × 10^−16^	47	*VAPA*, *NPM1*, *FLNC*, *TCF3*, *ABL1*, *RIPK1*, *MAP1B*, *CALR*, *DISC1*, *MAX*, *ICAM1*, *SPTBN1*, *REL*, *RPS19*, *RELA*, *RELB*, *LASP1*, *PRC1*, *SKI*, *AR*, *HNRNPU*, *CCNB1*, *CASK*, *MEN1*, *KIF20A*, *CFLAR*, *NEFM*, *IFIH1*, *AGO1*, *RNF4*, *AP1G1*, *EGR1*, *CBX5*, *PIK3R1*, *TLE1*, *NFKB2*, *LRP1*, *KMT2A*, *FGF13*, *TNFRSF1A*, *FKBP1A*, *CX3CL1*, *NOTCH1*, *SMAD2*, *CRTAP*, *KIF2C*, *NCKAP1L*
3	GO:0042802	Identical protein binding	1.82 × 10^−07^	30	*CALCOCO2*, *NPM1*, *MYD88*, *TCF3*, *RIPK1*, *ATG7*, *MAX*, *ETS1*, *IRF3*, *RPS19*, *RELA*, *PRC1*, *IFI16*, *IFIT3*, *STAT1*, *CSF1R*, *RNF4*, *CBX5*, *TLE1*, *KMT2A*, *BCL10*, *USP15*, *CDC42*, *FKBP1A*, *G6PD*, *PCM1*, *MAD2L1*, *SMAD2*, *COL1A1*, *MAF*
4	GO:0005102	Signaling receptor binding	1.55 × 10^−05^	29	*MYD88*, *ABL1*, *RPGRIP1L*, *RIPK1*, *CALR*, *CANX*, *ICAM1*, *BLNK*, *NCK1*, *CBL*, *AR*, *STAT1*, *CCNB1*, *CASK*, *CFLAR*, *SNW1*, *RNF4*, *CD86*, *PIK3R1*, *LRP1*, *FGF13*, *USP15*, CDC42, *FKBP1A*, *CX3CL1*, *HCK*, *NOTCH1*, SMAD2, *CXCL8*
5	GO:0046983	Protein dimerization activity	1.07 × 10^−06^	28	*CALCOCO2*, *VAPA*, *NPM1*, *TCF3*, *ATG7*, *MAX*, *BAD*, *ID2*, *IRF3*, *RPS19*, *RELA*, *AR*, *STAT1*, *CUL5*, *CFLAR*, *CSF1R*, *RNF4*, *CBX5*, *PIK3R1*, *KMT2A*, *BCL10*, *FKBP1A*, *G6PD*, *NOTCH1*, *MAD2L1*, *SMAD2*, *MAF*, *CYBA*
6	GO:0043565	Sequence-specific DNA binding	4.31 × 10^−06^	24	*TCF3*, *GATAD2A*, FOSB, *MAX*, *ETS1*, *IRF2*, *IRF3*, *REL*, *RELA*, *RELB*, *IFI16*, *AR*, *STAT1*, *NLRP3*, *MEN1*, *ORC2*, *AGO1*, *EGR1*, *NFKB2*, *KMT2A*, *NOTCH1*, *SMAD2*, *KIF2C*, *MAF*
7	GO:0003700	DNA-binding transcription factor activity	1.26 × 10^−04^	23	*TCF3*, *GATAD2A*, *FOSB*, *MAX*, *ETS1*, *IRF2*, *IRF3*, *REL*, *RELA*, *RELB*, *CBL*, *IFI16*, *AR*, *STAT1*, *RBCK1*, *GTF2I*, *RNF4*, *EGR1*, *NFKB2*, *KMT2A*, *NOTCH1*, *SMAD2*, *MAF*
8	GO:0019900	Kinase binding	2.14 × 10^−08^	22	*NPM1*, *TCF3*, *ABL1*, *BAD*, *BLNK*, *RPS19*, *RELA*, *NCK1*, *PRC1*, *SKI*, *CBL*, *CBLB*, *RBCK1*, *CCNB1*, *CCND2*, *KIF20A*, *LDB3*, *GTF2I*, *PIK3R1*, *BCL10, CDC42*, *MVP*
9	GO:0008092	Cytoskeletal protein binding	5.56 × 10^−06^	21	*VAPA*, *FLNC*, *ABL1*, *ANK1*, *MAP1B*, *DISC1*, *SPTBN1*, *TRPC6*, *SYNPO*, *RELA*, *NCK1*, *LASP1*, *PRC1*, *KIF20A*, *NEFM*, *LDB3*, *AP1G1*, *PARVA*, *FGF13*, *DMD*, *KIF2C*
10	GO:0044212	Transcription regulatory region DNA binding	1.25 × 10^−05^	20	*TCF3*, *GATAD2A*, *FOSB*, *MAX*, *IRF2*, *IRF3*, *REL*, *RELA*, *RELB*, *IFI16*, *AR*, *STAT1*, *HNRNPU*, *MEN1*, *AGO1*, *EGR1*, *NFKB2*, *KMT2A*, *NOTCH1*, *SMAD2*

**Table 4:** Top 10 cellular components enriched with their respective gene ontology terms and genes.

No.	GO ID	Name	*p* value	Count	Genes
1	GO:0044430	Cytoskeletal part	9.60 × 10^−09^	34	NPM1, RPGRIP1L, MAP1B, DISC1, SPTBN1, HAUS5, CENPU, ID2, XIAP, SYNPO, RELB, LASP1, PRC1, SKI, CCNB1, KIF20A, NEFM, ORC2, RIF1, CEP120, AMOT, ATP6V1D, CEP135, FGF13, BCL10, ESPL1, CDC42, G6PD, HSPA6, HCK, PCM1, MAD2L1, KIF2C, CYBA
2	GO:0030054	Cell junction	7.13 × 10^−09^	30	VAPA, NPM1, FLNC, RPGRIP1L, MAP1B, CALR, DISC1, ICAM1, PECAM1, TRPC6, IRF2, RPS19, SYNPO, NCK1, LASP1, DDB2, CASK, ARF6, RHOG, AMOT, PIK3R1, PARVA, LRP1, FGF13, DMD, CDC42, HCK, NOTCH1, APBB1IP, CYBA
3	GO:0015630	Microtubule cytoskeleton	6.75 × 10^−09^	29	VAPA, NPM1, RPGRIP1L, MAP1B, DISC1, HAUS5, CENPU, ID2, XIAP, RELB, PRC1, SKI, CCNB1, KIF20A, ORC2, RIF1, CEP120, AP1G1, ATP6V1D, CEP135, FGF13, BCL10, ESPL1, CDC42, G6PD, HSPA6, PCM1, MAD2L1, KIF2C
4	GO:0097458	Neuron part	5.23 × 10^−06^	29	ABL1, ANK1, MAP1B, ERC1, DISC1, CANX, MAX, SPTBN1, SYNPO, CBL, AR, STAT1, ASAP1, KHSRP, CASK, NEFM, GTF2I, PIK3R1, LRP1, CCR1, FGF13, DMD, TNFRSF1A, RAB2A, CDC42, FKBP1A, ALOX5, KPNA1, CYBA
5	GO:0043005	Neuron projection	4.02 × 10^−06^	25	ABL1, ANK1, MAP1B, DISC1, CANX, MAX, SPTBN1, SYNPO, CBL, AR, STAT1, ASAP1, KHSRP, CASK, NEFM, PIK3R1, LRP1, FGF13, DMD, TNFRSF1A, CDC42, FKBP1A, ALOX5, KPNA1, CYBA
6	GO:0005783	Endoplasmic reticulum	1.19 × 10^−03^	25	VAPA, COL6A2, ABL1, ANK1, COPG1, CALR, DISC1, CANX, NPC2, NCK1, BFAR, MAGT1, IFIT2, NLRP3, RHOG, FAF2, PIK3R1, EDEM1, RAB2A, CDC42, FKBP1A, NOTCH1, CRTAP, COL1A1, CYBA
7	GO:0098589	Membrane region	9.52 × 10^−05^	23	ANK1, RIPK1, ERC1, DISC1, ICAM1, SPTBN1, PECAM1, SYNPO, CBL, CBLB, PGK1, CASK, ARF6, CFLAR, TLR4, LRP1, AP1S2, BCL10, DMD, TNFRSF1A, HCK, NOTCH1, CYBA
8	GO:1902494	Catalytic complex	2.40 × 10^−05^	22	GATAD2A, RIPK1, CDK12, ERC1, DISC1, MAX, NCK1, MAGT1, DDB2, SAP30, RBCK1, MEN1, CCND2, CUL5, CFLAR, SNW1, AGO1, RNF4, CBX5, PIK3R1, KMT2A, CYBA
9	GO:0044427	Chromosomal part	1.71 × 10^−06^	21	PDS5B, TCF3, CENPL, CENPU, ID2, CENPK, AR, SAP30, STAT1, CCNB1, MEN1, ITGB3BP, CCND2, ORC2, RIF1, SNW1, CBX5, MAD2L1, SMAD2, KIF2C, MAF
10	GO:0005694	Chromosome	1.01 × 10^−05^	21	PDS5B, TCF3, CENPL, CENPU, ID2, CENPK, AR, SAP30, STAT1, CCNB1, MEN1, ITGB3BP, CCND2, ORC2, RIF1, SNW1, CBX5, MAD2L1, SMAD2, KIF2C, MAF

**Table 5:** Top 10 biological processes enriched with their respective gene ontology terms and genes.

No	GO ID	Name	*p* value	Count	Genes
1	GO:0012501	Programmed cell death	1.39 × 10^−15^	51	*NPM1*, *MYD88*, *ABL1*, *GATAD2A*, *RIPK1, ATG7*, *CALR*, *MAX*, *ICAM1*, *BAD*, *ETS1*, *XIAP*, *IRF3*, *RELA*, *NCK1*, *BFAR*, *SKI*, *CBL*, *IFI16*, *IFIT2*, *IFIT3*, *AR*, *STAT1*, *NLRP3*, *RBCK1*, *MEN1*, *ITGB3BP*, *ARF6*, *CUL5*, *CFLAR*, *SNW1*, *CSF1R*, *EGR1*, *PIK3R1*, *TLE1*, *TLR4*, *LRP1*, *KMT2A*, *FGF13*, *BCL10*, *TNFRSF1A*, *QKI*, *ESPL1*, *CDC42*, *CX3CL1*, *G6PD*, *HCK*, *NOTCH1*, *MAD2L1*, *KPNA1*, *NCKAP1L*
2	GO:0010628	Positive regulation of gene expression	1.76 × 10^−13^	47	*NPM1*, *MYD88*, *TCF3*, *RIPK1*, *CALR*, *ERC1*, *FOSB*, *ICAM1*, *ETS1*, *ID2*, *U2AF2*, *IRF2*, *REL*, *RELA*, *NCK1*, *RELB*, *SKI*, *IFI16*, *CENPK*, *AR*, *STAT1*, *NLRP3*, *RBCK1*, *HNRNPU*, *CCNB1*, *CASK*, *MEN1*, *CFLAR*, *RHOG*, *SNW1*, *AGO1*, *RNF4*, *EGR1*, *CD86*, *PIK3R1*, *TLE1*, *NFKB2*, *TLR4*, *KMT2A*, *BCL10*, *TNFRSF1A*, *QKI*, *CDC42*, *NOTCH1*, *SMAD2*, *COL1A1*, *MAF*
3	GO:0006955	Immune response	4.48 × 10^−15^	45	*CALCOCO2*, *MYD88*, *ABL1*, *RIPK1*, *ICAM1*, *BLNK*, *ETS1*, *XIAP*, *IRF2*, *IRF3*, *REL*, *RPS19*, *RELA*, *NCK1*, *SAMHD1*, *RELB*, *CBLB*, *IFI16*, *IFIT2*, *IFIT3*, *STAT1*, *NLRP3*, *RBCK1*, *ARF6*, *IFIH1*, *CSF1R*, *AP1G1*, *EGR1*, *CD86*, *PIK3R1*, *NFKB2*, *TLR4*, *CCR1*, *BCL10*, *TNFRSF1A*, *CDC42*, *FKBP1A*, *CX3CL1*, *HCK*, *NOTCH1*, *APBB1IP*, *CXCL8*, *COL1A1*, *NCKAP1L*, *CYBA*
4	GO:0065003	Protein-containing complex assembly	9.44 × 10^−13^	45	*NPM1*, *COL6A2*, *ABL1*, *RIPK1*, *MAP1B*, *CENPL*, *CALR*, *MAX*, *ICAM1*, *SPTBN1*, *PECAM1*, *HAUS5*, *BAD*, *CENPU*, *XIAP*, *RPS19*, *NCK1*, *SAMHD1*, *SKI*, *CENPK*, *DDB2*, *AR*, *NLRP3*, *CCNB1*, *MEN1*, *ITGB3BP*, *ARF6*, *AGO1*, *RNF4*, *TLE1*, *TLR4*, *PARVA*, *KMT2A*, *FGF13*, *BCL10*, *DMD*, *TNFRSF1A*, *CDC42*, *FKBP1A*, *CX3CL1*, *HCK*, *SMAD2*, *COL1A1*, *NCKAP1L*, *CYBA*
5	GO:0002682	Regulation of immune system process	1.19 × 10^−13^	42	*MYD88*, *TCF3*, *ABL1*, *RIPK1*, *ATG7*, *CALR*, *ICAM1*, *PECAM1*, *BAD*, *ETS1*, *ID2*, *XIAP*, *IRF3*, *RPS19*, *RELA*, *NCK1*, *SAMHD1*, *RELB*, *CBLB*, *IFI16*, *STAT1*, *NLRP3*, *RBCK1*, *ARF6*, *IFIH1*, *CSF1R*, *AP1G1*, *CD86*, *PIK3R1*, *TLR4*, *AP1S2*, *CCR1*, *BCL10*, *CDC42*, *FKBP1A*, *CX3CL1*, *HCK*, *NOTCH1*, *CXCL8*, *COL1A1*, *NCKAP1L*, *CYBA*
6	GO:0051254	Positive regulation of RNA metabolic process	7.81 × 10^−13^	42	*NPM1*, *MYD88*, *TCF3*, *RIPK1*, *ERC1*, *FOSB*, *ICAM1*, *ETS1*, *ID2*, *U2AF2*, *IRF2*, *REL*, *RELA*, *NCK1*, *RELB*, *SKI*, *IFI16*, *CENPK*, *AR*, *STAT1*, *NLRP3*, *RBCK1*, *CCNB1*, *CASK*, *MEN1*, *CFLAR*, *RHOG*, *SNW1*, *AGO1*, *RNF4*, *EGR1*, *CD86*, *PIK3R1*, *NFKB2*, *TLR4*, *KMT2A*, *BCL10*, *TNFRSF1A*, *NOTCH1*, *SMAD2*, *COL1A1*, *MAF*
7	GO:0080134	Regulation of response to stress	4.76 × 10^−11^	39	*CALCOCO2*, *NPM1*, *MYD88*, *NUP50*, *ABL1*, *RIPK1*, *ATG7*, *ETS1*, *U2AF2*, *XIAP*, *IRF3*, *RPS19*, *RELA*, *NCK1*, *SAMHD1*, *RELB*, *BFAR*, *IFI16*, *STAT1*, *NLRP3*, *CASK*, *MEN1*, *ARF6*, *IFIH1*, *RIF1*, *AP1G1*, *CD86*, *PIK3R1*, *ATP6V1D*, *TLR4*, *AP1S2*, *CCR1*, *BCL10*, *TNFRSF1A*, *EDEM1*, *CDC42*, *CX3CL1*, *HCK*, *CYBA*
8	GO:0051247	Positive regulation of protein metabolic process	8.67 × 10^−11^	39	*NPM1*, *MYD88*, *ABL1*, *RIPK1*, *ATG7*, *DISC1*, *ICAM1*, *PECAM1*, *TRPC6*, *BAD*, *XIAP*, *REL*, *RELA*, *NCK1*, *CBLB*, *IFI16*, *AR*, *STAT1*, *NLRP3*, *CCNB1*, *MEN1*, *CCND2*, *SNW1*, *CSF1R*, *CD86*, *PIK3R1*, *TLR4*, *KMT2A*, *CCR1*, *FGF13*, *BCL10*, *TNFRSF1A*, *EDEM1*, *ESPL1*, *CDC42*, *FKBP1A*, *CX3CL1*, *MAD2L1*, *NCKAP1L*
9	GO:0006952	Defence response	1.79 × 10^−10^	39	*CALCOCO2*, *MYD88*, *ABL1*, *RIPK1*, *ATG7*, *ICAM1*, *BLNK*, *ETS1*, *XIAP*, *IRF2*, *IRF3*, *REL*, *RPS19*, *RELA*, *SAMHD1*, *RELB*, *IFI16*, *IFIT2*, *IFIT3*, *STAT1*, *NLRP3*, *ARF6*, *IFIH1*, *CSF1R*, *AP1G1*, *EGR1*, *CD86*, *NFKB2*, *TLR4*, *AP1S2*, *CCR1*, *BCL10*, *TNFRSF1A*, *CX3CL1*, *HCK*, *ALOX5*, *NOTCH1*, *CXCL8*, *CYBA*
10	GO:1902531	Regulation of intracellular signal transduction	1.62 × 10^−09^	39	*VAPA*, *NPM1*, *MYD88*, *ABL1*, *RIPK1*, *CALR*, *CDK12*, *ICAM1*, *PECAM1*, *BAD*, *XIAP*, *IRF3*, *REL*, *RELA*, *NCK1*, *CBL*, *AR*, *STAT1*, *NLRP3*, *RBCK1*, *MEN1*, *ARF6*, *CFLAR*, *RHOG*, *CSF1R*, *AMOT*, *CD86*, *PIK3R1*, *TLE1*, *TLR4*, *CCR1*, *BCL10*, *DMD*, *TNFRSF1A*, *CDC42*, *FKBP1A*, *CX3CL1*, *NOTCH1*, *NLK*

Most of the gene sets are involved in immune- and inflammation-related pathways, like “innate immune system” (FDR = 3.21 × 10^−07^ *)*, “cytokine signaling in immune system” (FDR = 9.88 × 10^−08^), “adaptive immune system” (FDR = 4.84 × 10^−07^), “signaling by interleukins” (FDR = 3.04 × 10^−05^), and “NF kappa B signaling pathway” (FDR = 2.78 × 10^−12^), with gene counts ranging from 14 to 36. Details of the top 10 pathways enriched by the gene sets are represented in **Table 6**.

**Table 6:** Top 10 pathways enriched and number of genes.

No.	Pathway name	*p* value	Count	Hit in query list
1	Innate immune system	3.21E−07	36	*VAPA*, *MYD88*, *ABL1*, *RIPK1*, *ATG7*, *SPTBN1*, *PECAM1*, *BAD*, *IRF2*, *IRF3*, *NPC2*, *RELA*, *NCK1*, *RELB*, *MAGT1*, *IFI16*, *NLRP3*, *RHOG*, *IFIH1*, *AGO1*, *FAF2*, *CD86*, *PIK3R1*, *ATP6V1D*, *NFKB2*, *TLR4*, *BCL10*, *CDC42*, *MVP*, *HSPA6*, *HCK*, *GNS*, *ALOX5*, *APBB1IP*, *NCKAP1L*, *CYBA*
2	Cytokine signaling in immune system	9.88E−08	27	*IL10RA*, *MYD88*, *NUP50*, *ICAM1*, *SPTBN1*, *BLNK*, *IRF2*, *IRF3*, *RELA*, *SAMHD1*, *RELB*, *CBL*, *IFIT2*, *IFIT3*, *STAT1*, *CSF1R*, *EGR1*, *CD86*, *PIK3R1*, *NFKB2*, *CCR1*, *TNFRSF1A*, *HCK*, *ALOX5*, *APBB1IP*, *CXCL8*, *KPNA1*
3	Adaptive immune system	4.84E−07	27	*MYD88*, *ATG7*, *CALR*, *CANX*, *ICAM1*, *BAD*, *BLNK*, *REL*, *RELA*, *NCK1*, *DTX3L*, *CBL*, *CBLB*, *RBCK1*, *KIF20A*, *CUL5*, *AGO1*, *RNF4*, *AP1G1*, *CD86*, *PIK3R1*, *TLR4*, *AP1S2*, *BCL10*, *CDC42*, *KIF2C*, *CYBA*
4	Signaling by interleukins	3.04E−05	18	*IL10RA*, *MYD88*, *ICAM1*, *SPTBN1*, *BLNK*, *RELA*, *CBL*, *STAT1*, *CSF1R*, *CD86*, *PIK3R1*, *NFKB2*, *CCR1*, *TNFRSF1A*, *HCK*, *ALOX5*, *APBB1IP*, *CXCL8*
5	Signaling by Rho GTPases	1.06E−04	15	*ABL1*, *CENPL*, *CENPU*, *NCK1*, *PRC1*, *CENPK*, *AR*, *MEN1*, *ITGB3BP*, *RHOG*, *CDC42*, *MAD2L1*, *KIF2C*, *NCKAP1L*, *CYBA*
6	NF-κB signaling pathway	2.78E−12	14	*MYD88*, *RIPK1*, *ERC1*, *ICAM1*, *BLNK*, *XIAP*, *RELA*, *RELB*, *CFLAR*, *NFKB2*, *TLR4*, *BCL10*, *TNFRSF1A*, *CXCL8*
7	Neutrophil degranulation	1.37E−03	14	*VAPA*, *ATG7*, *PECAM1*, *NPC2*, *MAGT1*, *RHOG*, *FAF2*, *ATP6V1D*, *MVP*, *HSPA6*, *GNS*, *ALOX5*, *NCKAP1L*, *CYBA*
8	NOD-like receptor signaling pathway	4.94E−07	12	*MYD88*, *RIPK1*, *XIAP*, *IRF3*, *RELA*, *IFI16*, *STAT1*, *NLRP3*, *RBCK1*, *TLR4*, *CXCL8*, *CYBA*
9	Transcriptional mis-regulation in cancer	9.11E−07	12	*TCF3*, *MAX*, *ID2*, *REL*, *RELA*, *MEN1*, *CCND2*, *CSF1R*, *CD86*, *KMT2A*, *CXCL8*, *MAF*
10	Diseases of signal transduction	1.08E−03	12	*ATG7*, *BAD*, *CBL*, *STAT1*, *SNW1*, *CD86*, *PIK3R1*, *QKI*, *FKBP1A*, *NOTCH1*, *APBB1IP*, *SMAD2*

By analyzing the pathway interaction network, we observed that seed genes like *RIPK1*, *ATG7*, *TLR4*, *TNFRSF1A*, *KPNA1*, *CFLAR*, *SNW1*, *FOSB*, *PARVA*, *CX3CL1*, and TRPC6 interact with NK^PF^ category *RELA*, *RELB*, *NFKB2*, and *REL* in the normal condition and regulate the immune- and inflammatory-related pathways. But this connectivity among the genes are disrupted in the RA condition, leading to deregulation of the pathways. For ease of description, we termed these genes as “driver genes,” which include the NK^PF^ category as well. Notably, they are also involved in “signaling by interleukins,” “cytokine signaling in immune system,” “NOD-like receptor signaling pathway” (FDR = 4.93 × 10^−07^), “diseases of signal transduction” (FDR = 1 × 10^−04^), “MAPK signaling pathway” (FDR = 6.4 × 10^−05^), “Toll-like receptor signaling pathway” (FDR = 2.6 × 10^−9^), and “TNF signaling pathway” (FDR = 2.2 × 10^−5^). These pathways are also associated with inflammation and immune systems.

### GWAS Comparative Analysis

**Figure 9:**
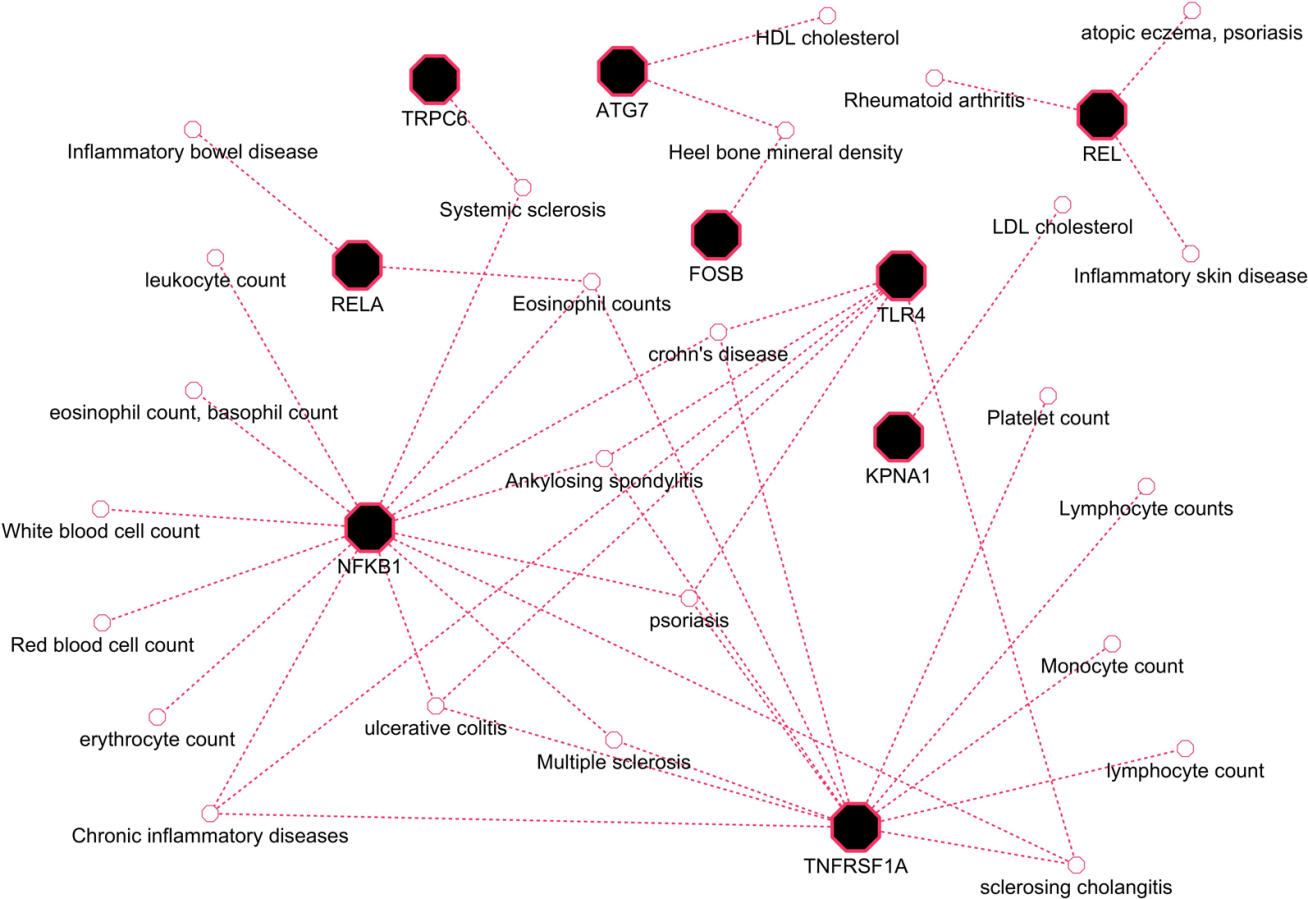
GWAS trait association to driver genes of RA.

Herein, an effort was made to relate susceptibility loci of inflammation-associated disease conditions in GWAS catalog to the driver genes of RA. We extracted the reported traits of these driver genes from GWAS catalog to detect their association to inflammatory conditions (**Supplementary File 2**, **Table S2**). There were 164 hits mapped to driver genes, but genes *SNW1* and *NFKB2* did not give any result. The GWAS-reported associations for genes of interest are presented in **Table 7**. We found a set of genes, *TNFRSF1A*, *TLR4*, *NFKB1*, *REL*, *RELA*, *ATG7*, *FOSB*, *KPNA1*, and *TRPC6*, significantly associated with RA. The GWAS overlap to these genes and their significant associated terms are represented in **Figure 9**. We also performed a literature survey for driver genes to understand whether they are previously linked to RA. This analysis by integrating GWAS studies also substantiates the role of identified genes have support from the genetic data. Outcome from this analysis provides new indications for clarifying the genetic mechanism of RA.

**Table 7:** The hits from GWAS analysis showing associations to inflammatory conditions.

No	Traits	Gene	P-value
1.	Ankylosing spondylitis	TNFRSF1A	3 x 10^−17^
2.	Crohn’s disease	TNFRSF1A	3 x 10^−17^
3.	Ulcerative colitis	TNFRSF1A	3 x 10^−17^
4.	Sclerosing cholangitis	TNFRSF1A	3 x 10^−17^
5.	Psoriasis	TNFRSF1A	3 x 10^−17^
6.	Ankylosing spondylitis	TLR4	3 x 10^−17^
7.	Crohn’s disease	TLR4	1 x 10^−8^
8.	Ulcerative colitis	TLR4	1 x 10^−8^
9.	Sclerosing cholangitis	TLR4	1 x 10^−8^
10.	Psoriasis	TLR4	1 x 10^−8^
11.	Ankylosing spondylitis	NFKB1	2 x 10^−18^
12.	Crohn’s disease	NFKB1	2 x 10^−18^
13.	Ulcerative colitis	NFKB1	2 x 10^−18^
14.	Sclerosing cholangitis	NFKB1	2 x 10^−18^
15.	Psoriasis	NFKB1	2 x 10^−18^
16.	Atopic eczema, psoriasis	REL	7 x 10^−9^
17.	Chronic inflammatory diseases	TNFRSF1A	3 x 10^−17^
18.	Chronic inflammatory diseases	TLR4	1 x 10^−8^
19.	Chronic inflammatory diseases	NFKB1	2 x 10^−18^
20.	Eosinophil count	TNFRSF1A	1 x 10^−20^
21.	Eosinophil count	RELA	2 x 10^−12^
22.	Eosinophil count	NFKB1	9 x 10^−12^
23.	Eosinophil count, basophil count	NFKB1	9 x 10^−11^
24.	Eosinophil counts	TNFRSF1A	1 x 10^−20^
25.	Eosinophil counts	RELA	2 x 10^−12^
26.	Eosinophil counts	NFKB1	9 x 10^−12^
27.	Erythrocyte count	NFKB1	3 x 10^−11^
28.	HDL cholesterol	ATG7	5 x 10^−8^
29.	Heel bone mineral density	ATG7	3 x 10^−12^
30.	Heel bone mineral density	FOSB	3 x 10^−12^
31.	Inflammatory bowel disease	RELA	4 x 10^−6^
32.	Inflammatory skin disease	REL	7 x 10^−9^
33.	LDL cholesterol	KPNA1	1 x 10^−8^
34.	LDL cholesterol levels	KPNA1	3 x 10^−8^
35.	Leukocyte count	NFKB1	7 x 10^−11^
36.	lymphocyte count	TNFRSF1A	1 x 10^−9^
37.	Lymphocyte counts	TNFRSF1A	1 x 10^−9^
38.	Monocyte count	TNFRSF1A	1 x 10^−16^
39.	Multiple sclerosis	TNFRSF1A	5 x 10^−6^
40.	Multiple sclerosis	NFKB1	1 x 10^−8^
41.	Platelet count	TNFRSF1A	5 x 10^−9^
42.	Red blood cell count	NFKB1	3 x 10^−11^
43.	Rheumatoid arthritis	REL	8 x 10^−7^
44.	Systemic sclerosis	TRPC6	4 x 10^−6^
45.	Systemic sclerosis	NFKB1	3 x 10^−7^
46.	White blood cell count	NFKB1	7 x 10^−11^

## Discussion

Classical gene expression techniques generally focus on identifying the single gene that shows a differential expression between two different conditions of interest. Although it is a valuable approach, it often fails to detect metabolic pathways, stress reactions, biological processes, and transcriptional regulations which arise from coordinated activities of a cluster of gene networks (Ghazalpour et al., 2005). Systems biology techniques try to unravel the multifaceted complex conditions or diseases, which usually will not develop due to the instabilities in a single gene function, but instead from changes in several pathways through various intra- and inter-relationships. In addition, different processes occurring inside the body are regulated by specific group of protein complexes. It is well known that in disease condition, protein interaction networks are often altered, stimulating diverse molecular events via a series of processes initiated by the dysregulated protein molecules in the biological network. Network biology provides a great platform to understand the biological mechanisms that can trigger a disease. Present study explores the ideas of correlation, co-expression, and semantic similarity integrated with network biology of genes to identify specific changes in RA condition. We used local and global parameters, DC and BC, respectively, to dissect the complex interactome—PPIM. The DC of a gene is the number of partners that are connected to that specific gene. On other hand, betweenness centrality, BC, of a node exerts control over other nodes which are functionally relevant in the network. The constructed network follows the characteristics of a scale-free network as the connectivity of nodes is in a heterogeneous distribution. The heterogeneous distribution of nodes represents a large number of nodes with few connectivities and few nodes have large number of interactions (Albert, 2005). An edge between the nodes can be a functional or physical connection, which can be a regulatory crosstalk, protein binding, or metabolic action (Barabasi et al., 2011). We observed significant variations in the expression of selected prioritized genes in both the healthy and affected tissues in RA. Initially, a complex network of significant genes from synovial tissue was created which was further disintegrated into a Regulator Allied Protein Interaction Network (RA^PIN^) based on hubs and bottlenecks. Hubs are considered as key features, and they point to crucial intersections among clusters of genes in a network such that the network is disordered when hub nodes are detached (Csermely et al., 2013). In the biological interactome RA^PIN^, essential genes are indicated as nodes with high connectivity or degree. Several research investigations have reported that genes involved in disease conditions have higher number of interactions or connectivity when compared to other genes not connected to the disease. We obtained 131 hubs with an average connectivity of 132.54 edges. This supports the significance of hub genes in the biological interactome (Khosravi et al., 2014).

Interestingly, *NFKB1*, *REL*, and *RELA* of the NK^PF^ group and genes like *TLR4*, *TNFRSF1A*, and *SNW1* of the seed gene set were found in hubs. Functional enrichment and GWAS analysis revealed that the aforesaid hub genes except *SNW1* were directly linked with RA. Thus, pinpointing hub genes in the biological interactome can provide improved understanding of disease pathogenesis. On other hand, betweenness centrality of a gene is the control of a gene that indicates the connectivity of other genes which are functionally significant in the interactome. Bottleneck genes show higher betweenness centrality and favor genes which are connected to compact networks instead of genes that are positioned inside the compact cluster (Yoon et al., 2006). *GRIN2B*, *KPNA1*, *PRKCG*, *CX3CL1*, *RIPK1*, *CFLAR*, *CALCOCO2*, *ATG7*, and *ITCH* of seed genes and *NFKB2* and *RELB* of the NK^PF^ category were present in the bottleneck group. The functional enrichment and GWAS analysis revealed that *NFKB2*, *RELB*, *CX3CL1*, *RIPK1*, and *ATG7* are associated with RA. The gene list which emerged after all filtering methods was considered as “driver genes.”

The established theory of network medicine is that primary interacting partners of disease-causing genes may play a role in related diseases. In addition, in a network of disease genes, the non-disease genes tend to have a higher tendency to interact with other disease genes (Csermely et al., 2013). We explored this hypothesis with diseases and pathways related to the driver genes.

*NFKB1*, *REL, RELA, TLR4*, *TNFRSF1A*, and *SNW1* act as a central hub in the network with high number functional partners. In the functional enrichment analysis and literature survey, *NFKB1*, *REL, RELA*, *TLR4*, and *TNFRSF1A* are functionally contributing to RA These genes are also involved in pathways of *innate immune system*, *cytokine signaling in immune system*, *NF kappa B signaling pathway*, *osteoclast differentiation*, *TNF signaling pathway*, etc. The role of NF-κB in RA and its potential to become a therapeutic target is extensively reported. Earlier study reveals the role of NF-κB and its significance to RA and inflammation (Simmonds and Foxwell, 2008). Other studies support the hypothesis that NF-κB triggers the pathogenesis of RA and plays a dominant role in eliciting chronic inflammation (Noort et al., 2015; Liu et al., 2017). The molecular stimulating event that initiates this process is not known, but may possibly contain molecules on the surface of T cell receptors and/or Toll-like receptor family ligands. These stimulate macrophage molecules in the synovium, resulting in the phosphorylation of the inhibitor of κB by the complex of IκB kinase that leads to destruction by the proteasome. This discharges NF-κB dimers such as p50/p65 that bring the expression of several pro-inflammatory chemokines and cytokines and results in inflammation and penetration of high amount of immune-related elements into the synovium. Inflammatory mediators, mainly TNF-α, stimulate cells in the synovium in a paracrine and autocrine manner, which is NF-κB-dependent. Fibroblasts trigger various NF-κB-induced genes by reacting to TNF-α or IL-1, together with chemokines, which results in more inflammatory infiltrates and matrix metalloproteinases that initiate joint destruction. Genes of NK^PF^ (family of NF-κB proteins) have a critical role in RA by regulating inflammation and immune system (Makarov, 2001; Feldmann et al., 2002; Roman-Blas and Jimenez, 2006; Noort et al., 2015).

Evidence for the role of another hub gene, *TNFRSF1A* in RA is extensive. Four GWAS in RA have strongly identified 12p13 as a susceptibility locus, where TNFRSF1A is mapped (Cornelis et al., 1998; Shiozawa et al., 1998; Jawaheer et al., 2001; Osorio et al., 2004). Mutations in the *TNFRSF1A* gene are dominantly inherited in TRAP syndrome (TRAPS), signifying the association of *TNFRSF1A* to auto-inflammatory diseases. The R92Q mutation in TNFRSF1A could be involved in RA, as was seen in 5.2% of 135 patients with early arthritis (Aksentijevich et al., 2001; Nowlan et al., 2006; Dieude et al., 2007; Mewar and Wilson, 2011).

Next, we considered the involvement of bottleneck genes (*RIPK1*, *ATG7*, *NFKB2*, *RELB*, *KPNA1*, *CFLAR*, and *CX3CL1*) in RA. More interestingly, two genes of the NK^PF^ category, *NFKB2* and *RELB*, are found in the bottleneck group. This implies that both hubs and bottlenecks comprised NK^PF^ gene category.

We identified *SNW1*, a hub gene, and *RIPK1* and *ATG7*, bottleneck genes, which are part of the NK^PF^ group. *RIPK1* and *ATG7* are also seen in RA pathways. *RIPK1* is co-expressed with *RELA* and is also connected to another RA-associated gene, *TLR4* (**Figure 7**). Recent study reveals that inhibition of *RIPK1* reduces the severity of the experimental autoimmune arthritis through osteoclastogenesis suppression (Jhun et al., 2019). The process of osteoclastogenesis is linked to the NF-κB activity. Activation of NF-κB is essential for the formation of osteoclasts (Boyce et al., 2015). There is supporting evidence for inactivating *RIPK1* to develop inflammatory response and reduce death of necroptotic cell *in vitro* and *in vivo*. A recent study reported *RIPK1* as a promising therapeutic target for the treatment of a wide range of human autoimmune and inflammatory diseases (Degterev et al., 2019). They also revealed that *RIPK1* is a significant mediator of inflammatory pathways (Degterev et al., 2019). This provides significant support to the network medicine theory that the primary interacting partners of disease genes are contributing to related diseases as well.

The ATG7 gene is co-expressed with RELB, another NKPF gene. This gene codes for E1-like stimulating enzyme that is essential for autophagy and to vacuole transport. Autophagy is a process of degradation whereby cells reutilize cytoplasmic constituents to produce energy. Dysregulation of autophagy pathway is implicated in the pathogenesis of RA. Autophagy controls apoptosis resistance, increases cell division in synovial fibroblasts, and endorses osteoclastogenesis leading to RA (Dai and Hu, 2016). Autophagy may contribute to RA development through multiple processes, e.g., in continuing autoimmune response by cytokine production, survival of inflammatory and autoreactive cells, and presentation of citrullinated antigens to the major histocompatibility complex (Foulquier et al., 2007; Vomero et al., 2018). Also, autophagy inhibits the development of autoimmunity process by removing intracellular pathogens and preserves immune cell homeostasis, with the regulation of immunological tolerance processes. In experimental mouse models of RA, autophagy inhibition decreased symptoms of bone destruction and the quantity of osteoclasts. In this context, drugs that can downregulate autophagy can be used to inhibit resorption of bone in RA patients (Lin et al., 2013).

One of the important findings in our study is the novel association of *SNW1* with *REL*. Till date, the role *SNW1* in RA is not reported. This gene becomes significant as it is a hub gene with more functional partners and a strong correlation with *REL*. Hubs specify significant connectivity among clusters in the biological interactome such that the biological interactions are disordered when hub nodes are detached (Khosravi et al., 2014). Genes involved in disease conditions have higher number of interactions or connectivity when compared to non-diseased genes (Csermely et al., 2013). More interestingly, a recent study has described the critical role of *SNW1* in the NF-κB pathway and reports *SNW1* as a novel transcriptional regulator of the NF-κB pathway. They also demonstrate that *SNW1* is indispensable for the transcriptional elongation of NF-κB target genes like interleukin 8 and tumor necrosis factor genes (Verma et al., 2019). Hence, *SNW1*’s role in RA can be considered as a novel finding which may act as potential biomarker or drug target for RA as they are found together with other key genes of RA.

Our approach, however, has certain limitations. First, as experimentally proven protein interactions are extracted using Bisogenet plugin, which utilizes interactions from various protein interaction databases, any interactions that are not included in the databases are missing in our work. Furthermore, there are insufficient evidences for few genes engaged in molecular functions or biological processes in gene ontology (GO). In order to overcome these limitations, we tried to include protein interaction based on co-expression. Overall, our research analysis has presented the effectiveness of linking gene expression with their functional relationships in the identification of RA candidate genes. Future experimental validation is required to demonstrate the direct or indirect involvement of the novel candidate genes uncovered in the current study in RA.

## Conclusion

This work systematically and scientifically outlines an integrated bioinformatics pipeline to find the most indispensable key signatures from the interactome Regulators Allied Protein Interaction Network (RA^PIN^). A detailed parametric downstream analysis based on biological insights highlights 11 candidate genes that can act as potential biomarkers or drug targets for RA. One of the remarkable highlights of this analysis is the identification of *SNW1* as potential biomarker for RA. Overall, our research analysis has presented the effectiveness of linking genetic expression with their functional relationship in the identification of RA candidate genes. By experimentally authenticating the results using *in vitro* and *in vivo* experiments, this can be further extended in order to pinpoint more selective therapeutic agents.

## Data Availability Statement

All datasets analyzed for this study are included in the article/**Supplementary Material**.

## Author Contributions

Conceptualization: JS, RE, NS, and MK. Data curation: AE, BB, MS, and HZ. Formal analysis: MA-S, NH, LC, KN, and MK. Funding acquisition: JS. Methodology: BB. Project administration: NA. Resources: MK. Software: BB. Supervision: RE, NS, and MK. Validation: HZ. Visualization: BB. Writing, Original draft: BB, NS, and MK. Writing, review and editing: JS, AE, MA-S, NA, MS, NH, KN, NH, RE, NS, LC, and MK.

## Funding

This work was supported by King Abdulaziz City for Science and Technology (KACST), Riyadh, Saudi Arabia, research grant no. AT-35-140.
