# Supplementary material for: Dissecting the Role of NF-κb Protein Family and Its Regulators in Rheumatoid Arthritis Using Weighted Gene Co-Expression Network
Source: Front Genet. 2019 Nov 20;10:1163. doi: 10.3389/fgene.2019.01163 (PMC6879671; doi:10.3389/fgene.2019.01163)
Supplement: Supplementary file 1 [file DataSheet_1.docx]

**Table S1:** The information about the patient samples used in the analysis.

| **No** | **Sample** | **Type** | **Tissue** |
| --- | --- | --- | --- |
|  | GSM2048265 | Healthy Control | Synovium |
|  | GSM2048266 | Healthy Control | Synovium |
|  | GSM2048267 | Healthy Control | Synovium |
|  | GSM2048268 | Healthy Control | Synovium |
|  | GSM2048269 | Healthy Control | Synovium |
|  | GSM2048270 | Healthy Control | Synovium |
|  | GSM2048271 | Healthy Control | Synovium |
|  | GSM2048272 | Rheumatoid Arthritis | Synovium |
|  | GSM2048273 | Rheumatoid Arthritis | Synovium |
|  | GSM2048274 | Rheumatoid Arthritis | Synovium |
|  | GSM2048275 | Rheumatoid Arthritis | Synovium |
|  | GSM2048276 | Rheumatoid Arthritis | Synovium |
|  | GSM2048277 | Rheumatoid Arthritis | Synovium |
|  | GSM2048278 | Rheumatoid Arthritis | Synovium |
|  | GSM2048279 | Rheumatoid Arthritis | Synovium |
|  | GSM2048280 | Rheumatoid Arthritis | Synovium |
|  | GSM2048281 | Rheumatoid Arthritis | Synovium |
|  | GSM2048282 | Rheumatoid Arthritis | Synovium |
|  | GSM2048283 | Rheumatoid Arthritis | Synovium |
|  | GSM2048284 | Rheumatoid Arthritis | Synovium |
|  | GSM2048285 | Rheumatoid Arthritis | Synovium |
|  | GSM2048286 | Rheumatoid Arthritis | Synovium |
|  | GSM2048287 | Rheumatoid Arthritis | Synovium |

Table S2 : Driver genes from GWAS catalog Associated with inflammatory disease

| **No** | **Traits** | **Gene** | **P-value** | **Accession** |
| --- | --- | --- | --- | --- |
|  | Adolescent idiopathic scoliosis | TLR4 | 1E-9 | GCST006287 |
|  | Adolescent idiopathic scoliosis | TRPC6 | 6E-10 | GCST006287 |
|  | Adverse response to chemotherapy (neutropenia/leucopenia) (all anthracycline-based drugs) | TNFRSF1A | 7E-6 | GCST002008 |
|  | Adverse response to chemotherapy (neutropenia/leucopenia) (epirubicin) | TNFRSF1A | 3E-7 | GCST001996 |
|  | age at menarche | TRPC6 | 7E-14 | GCST002541 |
|  | age-related hearing impairment | PARVA | 5E-9 | GCST002487 |
|  | Age-related hearing impairment (SNP x SNP interaction) | PARVA | 5E-9 | GCST002487 |
|  | albumin:globulin ratio measurement | NFKB1 | 1E-12 | GCST005987 |
|  | Albumin-globulin ratio | NFKB1 | 1E-12 | GCST005987 |
|  | Allergic rhinitis | NFKB1 | 1E-15 | GCST006409 |
|  | Allergic sensitization | NFKB1 | 2E-8 | GCST006408 |
|  | allergic sensitization measurement | NFKB1 | 2E-8 | GCST006408 |
|  | Alopecia areata | TNFRSF1A | 8E-6 | GCST004866 |
|  | Alzheimer's disease or family history of Alzheimer's disease | RELB | 5E-8 | GCST005922 |
|  | Alzheimer's disease, family history of Alzheimer’s disease | RELB | 5E-8 | GCST005922 |
|  | Ankylosing spondylitis | TNFRSF1A | 8E-10 | GCST005529 |
|  | ankylosing spondylitis, crohn's disease, psoriasis, sclerosing cholangitis, ulcerative colitis | NFKB1 | 2E-18 | GCST005537 |
|  | ankylosing spondylitis, crohn's disease, psoriasis, sclerosing cholangitis, ulcerative colitis | TLR4 | 1E-8 | GCST005537 |
|  | ankylosing spondylitis, crohn's disease, psoriasis, sclerosing cholangitis, ulcerative colitis | TNFRSF1A | 3E-17 | GCST005537 |
|  | asthma, response to diisocyanate | PARVA | 2E-6 | GCST002875 |
|  | atopic eczema, psoriasis | REL | 7E-9 | GCST002740 |
|  | balding measurement | PARVA | 2E-8 | GCST007038 |
|  | Balding type 1 | PARVA | 2E-8 | GCST007038 |
|  | behavioural disinhibition measurement | RELA | 3E-7 | GCST007621 |
|  | biliary liver cirrhosis | NFKB1 | 4E-12 | GCST001010 |
|  | biliary liver cirrhosis | TNFRSF1A | 2E-9 | GCST001010 |
|  | Blood protein levels | CX3CL1 | 1E-16 | GCST005806 |
|  | Blood protein levels | TLR4 | 2E-50 | GCST004365 |
|  | blood protein measurement | CX3CL1 | 1E-16 | GCST005806 |
|  | blood protein measurement | TLR4 | 3E-70 | GCST005806 |
|  | Breast cancer | CFLAR | 8E-8 | GCST004988 |
|  | breast carcinoma | CFLAR | 8E-8 | GCST004988 |
|  | Cardiovascular disease | ATG7 | 1E-10 | GCST007072 |
|  | Change in LVEF in response to paclitaxel and trastuzumab in HER2+ breast cancer | TRPC6 | 8E-6 | GCST004790 |
|  | chemerin measurement | ATG7 | 9E-7 | GCST004389 |
|  | Child Behaviour Checklist assessment | RIPK1 | 5E-7 | GCST002362 |
|  | Cholesterol, total | RELB | 9E-17 | GCST004209 |
|  | Chronic inflammatory diseases (ankylosing spondylitis, Crohn's disease, psoriasis, primary sclerosing cholangitis, ulcerative colitis) (pleiotropy) | NFKB1 | 2E-18 | GCST005537 |
|  | Chronic inflammatory diseases (ankylosing spondylitis, Crohn's disease, psoriasis, primary sclerosing cholangitis, ulcerative colitis) (pleiotropy) | TLR4 | 1E-8 | GCST005537 |
|  | Chronic inflammatory diseases (ankylosing spondylitis, Crohn's disease, psoriasis, primary sclerosing cholangitis, ulcerative colitis) (pleiotropy) | TNFRSF1A | 3E-17 | GCST005537 |
|  | Chronic lymphocytic leukemia | CFLAR | 5E-11 | GCST004146 |
|  | Chronotype | NFKB1 | 4E-8 | GCST004696 |
|  | circadian rhythm | NFKB1 | 4E-8 | GCST004696 |
|  | Circulating chemerin levels | ATG7 | 9E-7 | GCST004389 |
|  | colorectal adenoma, colorectal cancer | TRPC6 | 1E-9 | GCST007856 |
|  | Colorectal cancer | TRPC6 | 3E-6 | GCST005150 |
|  | Colorectal cancer or advanced adenoma | TRPC6 | 1E-9 | GCST007856 |
|  | cytotoxicity measurement, response to triptolide | CFLAR | 3E-7 | GCST003008 |
|  | Depression | ATG7 | 5E-8 | GCST006477 |
|  | Depression | TLR4 | 1E-21 | GCST007342 |
|  | depressive symptom measurement, drug use measurement, SSRI use measurement | PARVA | 8E-7 | GCST002755 |
|  | depressive symptom measurement, stressful life event measurement | RIPK1 | 4E-6 | GCST007413 |
|  | Depressive symptoms (SSRI exposure interaction) | PARVA | 8E-7 | GCST002755 |
|  | Depressive symptoms x independent stressful life events interaction (2df test) | RIPK1 | 4E-6 | GCST007413 |
|  | Depressive symptoms x independent stressful life events interaction (1df test) | RIPK1 | 3E-6 | GCST007412 |
|  | Diastolic blood pressure | TRPC6 | 6E-9 | GCST006630 |
|  | Diisocyanate-induced asthma | PARVA | 2E-6 | GCST002875 |
|  | Eczema | NFKB1 | 5E-14 | GCST007075 |
|  | Eczema | REL | 3E-9 | GCST007075 |
|  | Educational attainment (MTAG) | KPNA1 | 2E-10 | GCST006571 |
|  | Educational attainment (years of education) | KPNA1 | 3E-8 | GCST006442 |
|  | eosinophil count | NFKB1 | 9E-12 | GCST004606 |
|  | eosinophil count | RELA | 2E-12 | GCST007065 |
|  | eosinophil count | TNFRSF1A | 1E-20 | GCST007065 |
|  | eosinophil count, basophil count | NFKB1 | 9E-11 | GCST004624 |
|  | Eosinophil counts | NFKB1 | 9E-12 | GCST004606 |
|  | Eosinophil counts | RELA | 2E-12 | GCST007065 |
|  | Eosinophil counts | TNFRSF1A | 1E-20 | GCST007065 |
|  | Eosinophil percentage of granulocytes | NFKB1 | 1E-10 | GCST004617 |
|  | Eosinophil percentage of white cells | NFKB1 | 6E-9 | GCST004600 |
|  | Epstein-Barr virus copy number in lymphoblastoid cell lines | PARVA | 6E-6 | GCST004735 |
|  | Epstein-Barr virus infection | PARVA | 6E-6 | GCST004735 |
|  | erythrocyte count | NFKB1 | 3E-11 | GCST007069 |
|  | Facial depth | TRPC6 | 5E-8 | GCST003646 |
|  | facial depth measurement | TRPC6 | 5E-8 | GCST003646 |
|  | family history of Alzheimer’s disease | RELB | 2E-26 | GCST005921 |
|  | Family history of Alzheimer's disease | RELB | 2E-26 | GCST005921 |
|  | General risk tolerance (MTAG) | TRPC6 | 9E-9 | GCST007325 |
|  | HDL cholesterol | ATG7 | 5E-8 | GCST002223 |
|  | Heel bone mineral density | ATG7 | 3E-12 | GCST006288 |
|  | Heel bone mineral density | FOSB | 3E-12 | GCST006288 |
|  | high density lipoprotein cholesterol measurement | ATG7 | 5E-8 | GCST002223 |
|  | Highest math class taken (MTAG) | KPNA1 | 2E-9 | GCST006568 |
|  | Hypothyroidism | NFKB1 | 1E-11 | GCST007073 |
|  | Idiopathic osteonecrosis of the femoral head | RIPK1 | 5E-6 | GCST003918 |
|  | Inflammatory bowel disease | RELA | 4E-6 | GCST003043 |
|  | Inflammatory skin disease | REL | 7E-9 | GCST002740 |
|  | LDL cholesterol | KPNA1 | 1E-8 | GCST006612 |
|  | LDL cholesterol levels | KPNA1 | 3E-8 | GCST004233 |
|  | left ventricular diastolic function measurement, response to paclitaxel, response to trastuzumab | TRPC6 | 8E-6 | GCST004790 |
|  | leukocyte count | NFKB1 | 7E-11 | GCST007070 |
|  | low density lipoprotein cholesterol measurement | KPNA1 | 1E-8 | GCST006612 |
|  | Lung adenocarcinoma | PARVA | 3E-6 | GCST004744 |
|  | Lung adenocarcinoma | TRPC6 | 9E-6 | GCST004744 |
|  | lymphocyte count | TNFRSF1A | 1E-9 | GCST004627 |
|  | Lymphocyte counts | TNFRSF1A | 1E-9 | GCST004627 |
|  | Lymphocyte percentage of white cells | NFKB1 | 2E-21 | GCST004632 |
|  | Male-pattern baldness | PARVA | 1E-9 | GCST007020 |
|  | mathematical ability | KPNA1 | 2E-9 | GCST006568 |
|  | Mean corpuscular hemoglobin | KPNA1 | 1E-10 | GCST007068 |
|  | Menarche (age at onset) | TRPC6 | 7E-14 | GCST002541 |
|  | Monocyte count | TNFRSF1A | 1E-16 | GCST004625 |
|  | Monocyte percentage of white cells | NFKB1 | 1E-9 | GCST004609 |
|  | Mortality in heart failure | PARVA | 7E-6 | GCST000661 |
|  | mortality, heart failure | PARVA | 7E-6 | GCST000661 |
|  | Mouth ulcers | NFKB1 | 6E-11 | GCST007839 |
|  | Multiple sclerosis | NFKB1 | 1E-8 | GCST005531 |
|  | Multiple sclerosis | TNFRSF1A | 5E-6 | GCST000424 |
|  | nausea and vomiting of pregnancy severity measurement | TRPC6 | 2E-12 | GCST005929 |
|  | Neuroticism | TLR4 | 2E-10 | GCST006476 |
|  | neuroticism measurement | TLR4 | 2E-10 | GCST006476 |
|  | Neutrophil percentage of granulocytes | NFKB1 | 5E-9 | GCST004623 |
|  | Neutrophil percentage of white cells | NFKB1 | 1E-14 | GCST004633 |
|  | Non-albumin protein levels | NFKB1 | 1E-15 | GCST005990 |
|  | Non-melanoma skin cancer | ATG7 | 7E-6 | GCST005896 |
|  | non-melanoma skin carcinoma | ATG7 | 7E-6 | GCST005896 |
|  | omega-6 polyunsaturated fatty acid measurement | TLR4 | 1E-6 | GCST002444 |
|  | oral ulcer | NFKB1 | 6E-11 | GCST007839 |
|  | Plasma omega-6 polyunsaturated fatty acid levels (dihomo-gamma-linolenic acid) | TLR4 | 1E-6 | GCST002444 |
|  | Platelet count | TNFRSF1A | 5E-9 | GCST004603 |
|  | Plateletcrit | TNFRSF1A | 2E-16 | GCST004607 |
|  | Pneumonia | TNFRSF1A | 1E-6 | GCST005009 |
|  | Positive affect | ATG7 | 3E-6 | GCST003768 |
|  | Preschool internalizing problems | RIPK1 | 5E-7 | GCST002362 |
|  | Primary biliary cholangitis | NFKB1 | 2E-10 | GCST004302 |
|  | Primary biliary cholangitis | TNFRSF1A | 4E-9 | GCST004302 |
|  | Primary biliary cirrhosis | NFKB1 | 8E-14 | GCST005581 |
|  | Primary biliary cirrhosis | TNFRSF1A | 1E-14 | GCST005581 |
|  | Pulse pressure | ATG7 | 2E-12 | GCST007269 |
|  | pulse pressure measurement | ATG7 | 2E-12 | GCST007269 |
|  | Red blood cell count | NFKB1 | 3E-11 | GCST007069 |
|  | response to anthracycline-based chemotherapy, response to antineoplastic agent | TNFRSF1A | 7E-6 | GCST002008 |
|  | response to epirubicin, response to antineoplastic agent | TNFRSF1A | 3E-7 | GCST001996 |
|  | Rheumatoid arthritis | REL | 8E-7 | GCST000679 |
|  | Rheumatoid arthritis (ACPA-positive) | REL | 1E-11 | GCST005568 |
|  | risk-taking behaviour | TRPC6 | 9E-9 | GCST007325 |
|  | Scarlet fever | NFKB1 | 4E-6 | GCST005008 |
|  | Schizophrenia (treatment resistant) | NFKB1 | 2E-7 | GCST001458 |
|  | self reported educational attainment | KPNA1 | 2E-10 | GCST006571 |
|  | Sensation seeking | RELA | 3E-7 | GCST007621 |
|  | serum non-albumin protein measurement | NFKB1 | 1E-15 | GCST005990 |
|  | Severity of nausea and vomiting of pregnancy | TRPC6 | 2E-12 | GCST005929 |
|  | Sleep duration | TRPC6 | 2E-7 | GCST007561 |
|  | Small vessel stroke | ATG7 | 3E-6 | GCST005057 |
|  | Sum eosinophil basophil counts | NFKB1 | 9E-11 | GCST004624 |
|  | susceptibility to pneumonia measurement | TNFRSF1A | 1E-6 | GCST005009 |
|  | susceptibility to scarlet fever measurement | NFKB1 | 4E-6 | GCST005008 |
|  | systemic scleroderma | NFKB1 | 3E-7 | GCST006493 |
|  | systemic scleroderma | TRPC6 | 4E-6 | GCST005336 |
|  | Systemic sclerosis | NFKB1 | 3E-7 | GCST006493 |
|  | Systemic sclerosis | TRPC6 | 4E-6 | GCST005336 |
|  | Systolic blood pressure | ATG7 | 2E-8 | GCST006259 |
|  | toll-like receptor 4:Lymphocyte antigen 96 complex measurement | TLR4 | 2E-50 | GCST004365 |
|  | Tonsillectomy | NFKB1 | 5E-14 | GCST003995 |
|  | tonsillectomy risk measurement | NFKB1 | 5E-14 | GCST003995 |
|  | total cholesterol measurement | RELB | 9E-17 | GCST004209 |
|  | treatment refractory schizophrenia | NFKB1 | 2E-7 | GCST001458 |
|  | Triptolide cytotoxicity | CFLAR | 3E-7 | GCST003008 |
|  | Ulcerative colitis | NFKB1 | 5E-14 | GCST003045 |
|  | unipolar depression | ATG7 | 5E-8 | GCST006477 |
|  | unipolar depression | TLR4 | 1E-21 | GCST007342 |
|  | Waist-hip ratio | ATG7 | 2E-10 | GCST007067 |
|  | wellbeing measurement | ATG7 | 3E-6 | GCST003768 |
|  | White blood cell count | NFKB1 | 7E-11 | GCST007070 |
