# Supplementary material for: Dissecting the Role of NF-κb Protein Family and Its Regulators in Rheumatoid Arthritis Using Weighted Gene Co-Expression Network
Source: Front Genet. 2019 Nov 20;10:1163. doi: 10.3389/fgene.2019.01163 (PMC6879671; doi:10.3389/fgene.2019.01163)
Supplement: Supplementary file 2 [file Presentation_1.pdf]

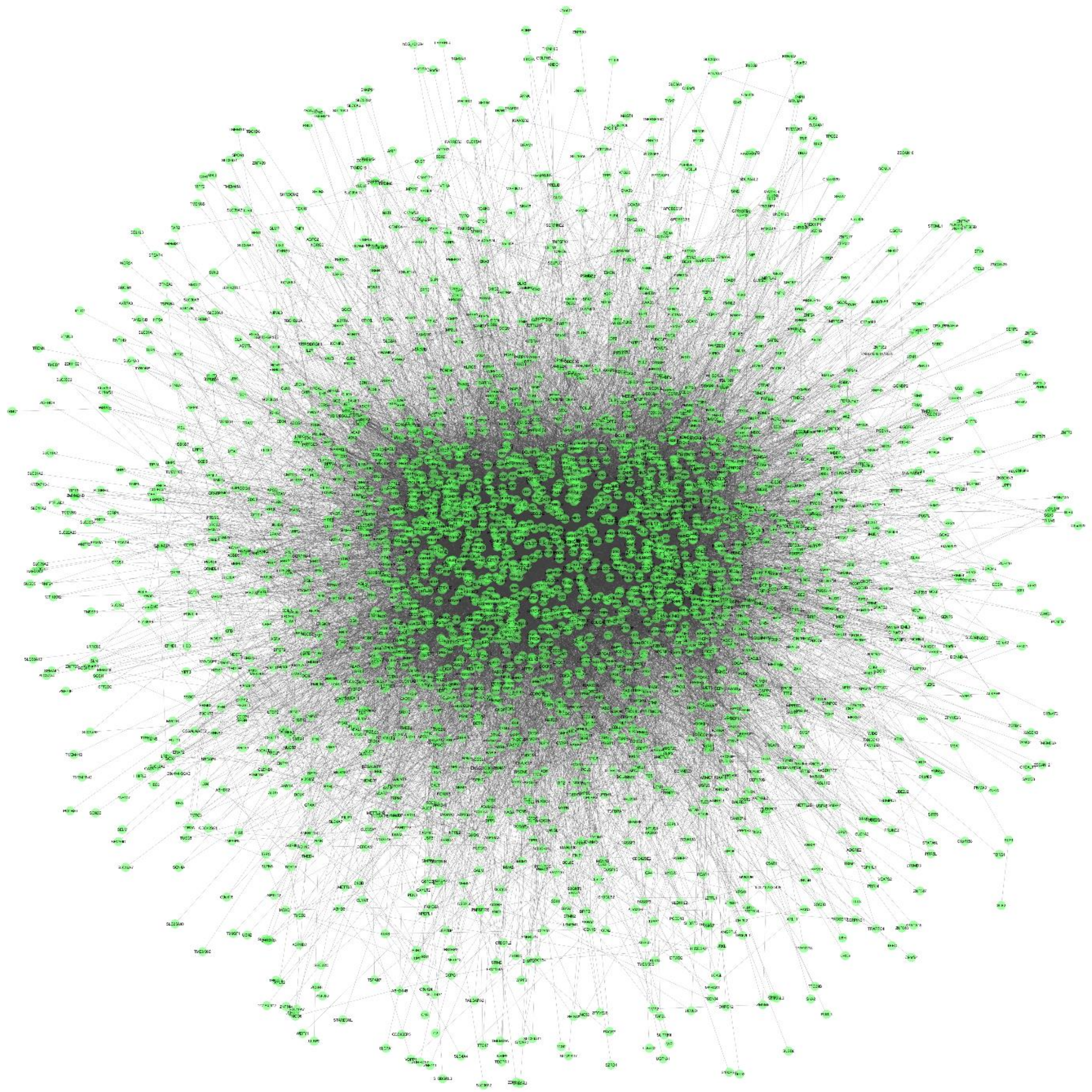

S-Figure. 1. The constructed PPIM consisting of 2742 nodes and 37032 edges with 13.51 average edge-node ratio

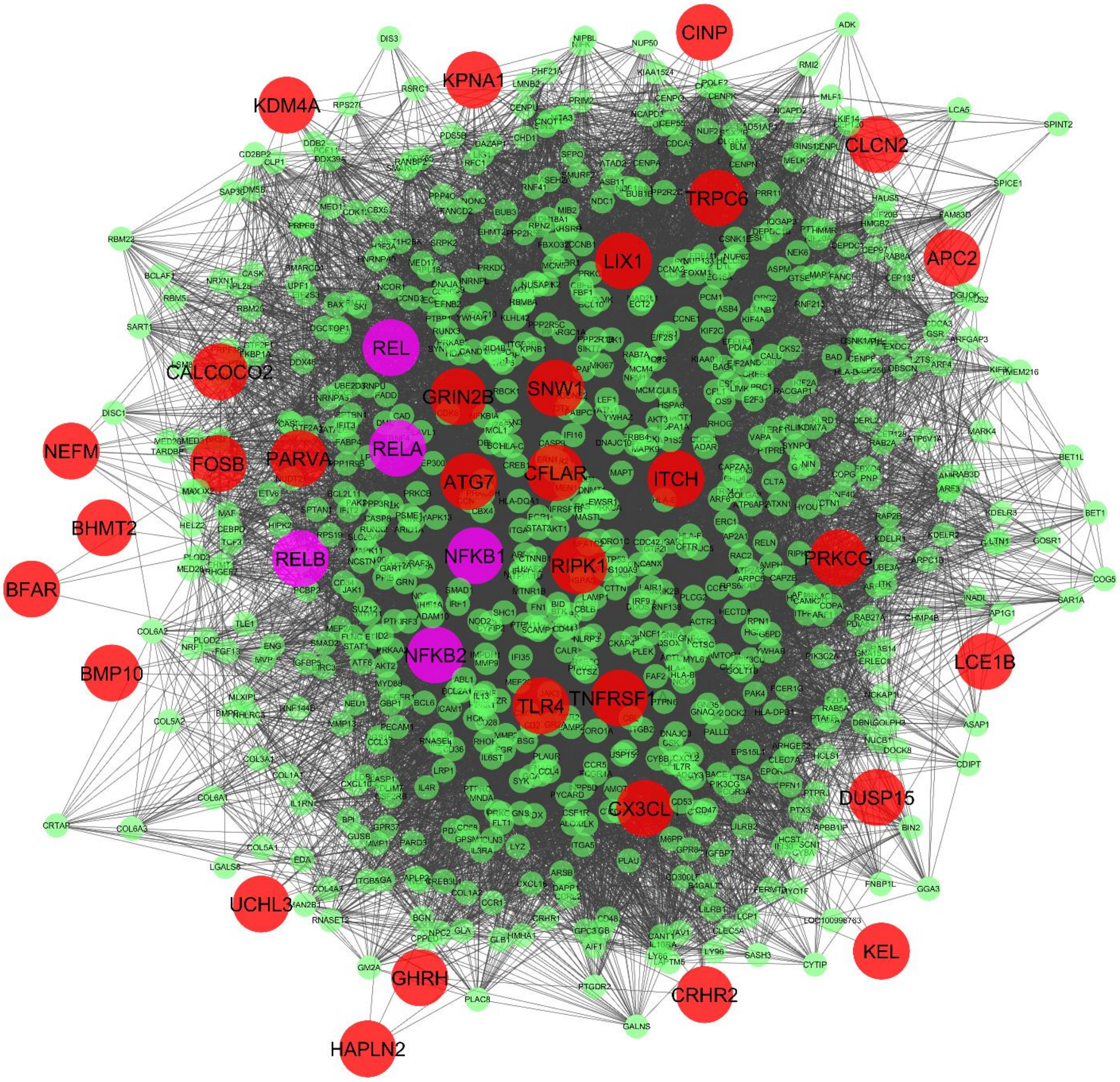

S-Figure. 2. The constructed RA^{PIN} from the interactions among the 801 genes extracted from main PPIM
